# SHP2 mutations induce precocious gliogenesis of Noonan syndrome-derived iPSCs during neural development in vitro

**DOI:** 10.1186/s13287-020-01709-4

**Published:** 2020-06-03

**Authors:** Younghee Ju, Jun Sung Park, Daejeong Kim, Bumsoo Kim, Jeong Ho Lee, Yoonkey Nam, Han-Wook Yoo, Beom Hee Lee, Yong-Mahn Han

**Affiliations:** 1 Department of Biological Sciences, KAIST, Daejeon, 34141 Republic of Korea; 2 Graduate School of Medical Science and Engineering, KAIST, Daejeon, 34141 Republic of Korea; 3 Department of Bio and Brain Engineering, KAIST, Daejeon, 34141 Republic of Korea; 4 Department of Pediatrics, Asan Medical Center Children’s Hospital, University of Ulsan College of Medicine, Seoul, 05505 Republic of Korea

**Keywords:** Noonan syndrome, Induced pluripotent stem cells, SHP2 mutations, Neural development, Gliogenesis, Cerebral organoids

## Abstract

**Background:**

Noonan syndrome (NS) is a developmental disorder caused by mutations of Src homology 2 domain-containing protein tyrosine phosphatase 2 (SHP2). Although NS patients have diverse neurological manifestations, the mechanisms underlying the involvement of SHP2 mutations in neurological dysfunction remain elusive.

**Methods:**

Induced pluripotent stem cells generated from dermal fibroblasts of three NS-patients (NS-iPSCs) differentiated to the neural cells by using two different culture systems, 2D- and 3D-cultured systems in vitro.

**Results:**

Here we represent that SHP2 mutations cause aberrant neural development. The NS-iPSCs exhibited impaired development of EBs in which BMP and TGF-β signalings were activated. Defective early neuroectodermal development of NS-iPSCs recovered by inhibition of both signalings and further differentiated into NPCs. Intriguingly, neural cells developed from NS-NPCs exhibited abundancy of the glial cells, neurites of neuronal cells, and low electrophysiological property. Those aberrant phenotypes were also detected in NS-cerebral organoids. SHP2 inhibition in the NS-NPCs and NS-cerebral organoids ameliorated those anomalies such as biased glial differentiation and low neural activity.

**Conclusion:**

Our findings demonstrate that SHP2 mutations contribute to precocious gliogenesis in NS-iPSCs during neural development in vitro.

## Background

Noonan syndrome (NS, OMIM 163950) is an autosomal genetic disorder characterized by distinctive craniofacial features, short stature, congenital cardiac anomalies, developmental delays, and variable neurocognitive impairments [1–4]. NS is caused by mutations of genes which encode a variety of signaling molecules related to the RAS-MAPK pathway, including *PTPN11* [5], *SOS1* [6, 7], *RAF1* [8, 9], and *RIT1* [10, 11]. Recently, it has been rarely detected in mutations of *KRAS* [12, 13], *NRAS* [14], *LZTR1* [15], *MRAS* [16, 17], and *RRAS2* [18, 19]. Among the various mutations, the *PTPN11* mutations which encodes Src homology 2 domain-containing protein tyrosine phosphatase 2 (SHP2) are most frequently occurring in NS patients [5, 20, 21]. Besides diverse manifestations, including short stature, craniofacial malformations, cardiac defects, and hematological abnormalities, an imbalance of cortical cell fates between neurons and glia is observed in Shp2 ^D61G/+^ mice with activated Ras-Mapk signaling and inhibited Jak-Stat3 signaling [22]. Despite increment of oligodendrocyte progenitor cells in the embryonic and postnatal brain, the Q79R-Shp2;Olig2^cre/+^ mice display abnormal myelination and fewer myelinated axons in the white matter compared with the wild-type (WT) mice [23, 24]. Knock-in mice expressing NS-associated mutations of *Ptpn11* represent an enhanced baseline excitatory synaptic function via the hyperactivation of Erk signaling, and they also show hippocampus-dependent impairments in spatial learning and long-term potentiation [25]. Clinical reports demonstrate that some NS patients with *PTPN11* mutations have low-grade glial tumors, including dysembryoplastic neuroepithelial tumors and pilocytic astrocytomas, that p-ERK level is elevated and glial fibrillary acidic protein (GFAP) is strongly expressed [26–28]. Nonetheless, how *PTPN11* mutations influence the cell fate during neural development is poorly understood in humans.

Furthermore, NS patients usually have a slight downward shift in cognitive capabilities, and their mean intelligence quotient (IQ) scores cluster around the low-to-average range [29]. Other neurological manifestations of NS patients include motor or cognitive developmental delays, learning disabilities, socialization problems, attention-deficit hyperactivity disorder (ADHD), and autism aspects [29–36]. Recently, modeling other rasopathies such as Costello syndrome (CS) and cardio-facio cutaneous (CFC) syndrome using human induced pluripotent stem cells (iPSCs) demonstrates neurodevelopmental abnormalities in vitro [37–40]. However, modeling of the neurological dysfunctions of NS patients has not been reported. In this context, cellular modeling using patient-specific iPSCs may provide insights on the neurological defects of NS patients.

Although it is commonly reproducible for the differentiation of iPSCs to neural cells, the 2-dimensional (2D) culture system has ultimate limitations to explore complexity, organization, and physiological environment of 3D-structured brain [41]. Cerebral organoids derived from human pluripotent stem cells are useful to study human brain development and neurological disease processes [42]. The brain organoids have multiple layers, various neural subtypes, and electrophysiological property, which partially recapitulate cortical development of a human brain in a dish [43–45]. Modeling neurodevelopmental disorders such as microcephaly and autism has been suggested in cerebral organoids derived from human iPSCs [46]. Cerebral organoids with heterozygous truncating mutation of *CDK5RAP2* gene show smaller size of organoids, reduction of early neural progenitors, and precocious neuronal differentiation as compared to control organoids, thereby demonstrating modeling of microcephaly [42]. Therefore, disease modeling using cerebral organoids is a valuable system to study neurological impairments of NS patients.

Here, NS-iPSCs exhibited various defects in the developmental process via embryoid bodies (EBs) formation, neural rosettes (NRs), and further neural differentiation. The developmental competence in defective NS-EBs reoccurred by the inhibition of the BMP and TGF-β pathways. Recovered NS-EBs normally developed to NRs and neural precursor cells (NPCs) despite the enhanced activities of SHP2 and ERK. Interestingly, NS-neural cells further differentiated in vitro exhibited imbalanced composition between neuronal and glial cells as compared with WT-neural cells. Additionally, NS-neural cells had shortened dendritic and axonal lengths. SHP2 inhibition rescued the biased development of glial cells and extended the shortened dendritic and axonal lengths in NS-neural cells. Electrophysiological reduction of NS-neural cells partially alleviated in terms of the number of extracellular spikes and spike frequency by SHP2 inhibition. NS-cerebral organoids also displayed enriched glial cells and decreased the number of spontaneous extracellular spikes. Those aberrant phenotypes in NS-cerebral organoids ameliorated to some extent by SHP2 inhibition. Our findings give a clue that the neurological dysfunctions of NS patients may be responsible for the neurodevelopmental impairments, including precocious gliogenesis, shortened neurites, and low electrophysiological property.

## Materials and methods

### Culture of human induced pluripotent stem cells

The human induced pluripotent stem cells (hiPSCs) used in the experiment were maintained on Mitomycin C (MMC, AG Scientific, San Diego, CA, USA)-treated mouse embryonic fibroblasts in hiPSC medium at 37 °C and 5% CO_2_ in air. The hiPSC medium consisted of Dulbecco’s modified Eagle medium (DMEM)/F-12 supplemented with 20% Knockout Serum Replacement (KSR), 1% nonessential amino acid, 1% penicillin-streptomycin (Invitrogen, Carlsbad, CA, USA), 0.1 mM β-mercaptoethanol, 1 mM L-glutamine (Sigma-Aldrich, St. Louis, Missouri, USA), and 10 ng/ml bFGF (R&D systems, Minneapolis, MN, USA). The medium was changed daily. The hiPSCs were passaged at an interval of 5 or 6 days.

### Generation of Noonan syndrome-iPSCs

NS-iPSCs were generated from dermal fibroblasts of the NS patients by ectopic expression of Yamanaka’s factors as previously described [47]. NS-fibroblasts were plated at a density of 1 × 10^5^ cells per 35 mm dish (BD Falcon, Franklin Lakes, NJ, USA) and then infected with the retroviruses mixed with 8 mg/ml polybrene (Hexadimethrine bromide, Sigma Aldrich) in DMEM containing 10% FBS, 1% nonessential amino acid, and 1% penicillin-streptomycin (Invitrogen) for 24 h. Then, the retroviruses were removed by replacing with fresh medium. After reaching confluence, the infected NS-fibroblasts were dissociated by treatment with 0.25% trypsin/EDTA (Invitrogen) for 2 min and then harvested. These cells were placed on MMC-MEFs with a density of 10,000–15,000 cells per each 35 mm dish. The next day, the medium was replaced with hiPSC medium supplemented with 10 ng/ml bFGF. The medium was changed daily. After 2 weeks of culture, putative colonies were mechanically picked up and then transferred onto fresh MMC-MEFs. Among four to eight iPSC-clones generated from respective three patients (NS1, NS2, and NS3), two clones (NS1, NS1-1, and NS1-2; NS2, NS2-1, and NS2-2; NS3, NS3-1, and NS3-2) were subjected to this study. A wild-type (WT)-iPSC line generated from human foreskin fibroblasts [48] was used as a control group.

### Karyotype analysis

The NS-iPSCs were maintained on the MMC-treated MEF for 5 days in a T75 flask (SPL Life Sciences Co.). The karyotype analysis was performed by Gendix (Seoul, Korea).

### Confirmation of *PTPN11* mutation

Genomic DNA (gDNA) of the hiPSCs and NS-dermal fibroblasts was isolated using a G-DEXTMIIC Genomic extraction kit (Intron Biotechnology, Inc., Seongnam, Korea). The mutation region of *PTPN11* was amplified via PCR with specific primers (forward: TTTTCCTGAAGCAGTCCAG; reverse: ATCCGCCAAAAGTCATTCAC). Target sequences were analyzed by Solgent Co., Ltd. (Daejeon, Korea).

### Differentiation of hiPSCs into neural cells

The neural differentiation of hiPSCs was performed as previously reported [49, 50]. Briefly, a single hiPSC colony was mechanically divided into several clumps and detached by treatment with 10 mg/ml collagenase IV (Invitrogen). Detached hiPSC clumps were transferred to low-attachment dishes (SPL Life sciences Co., Pocheon, Korea) and then cultured in DMEM/F12 containing 20% KSR, 1% nonessential amino acids, 1% penicillin and streptomycin (Invitrogen), 1 mM L-glutamine, and 0.1 mM ß-mercaptoethanol (Sigma-Aldrich). hiPSC-derived EBs attached on a Matrigel-coated cell culture dish (BD Falcon) were cultured in the NR/NPC medium for 8–10 days. The NR/NPC medium consisted of DMEM/F12, 0.5× B-27 supplement, 1× N2 supplement (Invitrogen), 1% nonessential amino acid, 1% penicillin-streptomycin (Invitrogen), 0.1 mM β-mercaptoethanol, 1 mM L-glutamine (Sigma Aldrich), and 20 ng/ml bFGF (R&D systems). The NR/NPC medium was changed daily. NRs were mechanically picked up, transferred to 35 mm petri dishes (SPL Life sciences Co.), and incubated in NR/NPC medium for 1–2 weeks until neurospheres were formed. At intervals of 2–3 days during NPC differentiation, neurospheres (NPCs) were mechanically triturated by gentle pipetting, resuspended in NR/NPC medium on 35 mm petri dishes, and incubated to expand the number of NPCs.

Prior to neural differentiation, the culture dishes were coated with 15 μg/ml poly-L-ornithine (PLO, Sigma-Aldrich) overnight and then coated with 2 μg/ml Laminin (BD Biosciences) overnight. The NPCs were treated with Accutase (eBioscience Inc., San Diego, CA, USA) at 37 °C for 20–30 min until dissociation. The collected cells were plated at a concentration of approximately 125,000 cells/cm^2^ and incubated in the NR/NPC medium on the PLO/Laminin-coated dish for 3–4 days. For differentiation into neural cells, confluent NPCs were cultured in the neurobasal medium (Invitrogen) supplemented with 1× N2 supplement, 0.5× B-27 supplement (Invitrogen), and 0.5 mM L-glutamine (Sigma Aldrich) for 21–23 days.

### Chemical treatments

The following chemicals were added to the EB medium during EB formation of hiPSCs: 10 μM SB431542 (SB, Abcam, Cambridge, MA, USA), 5 μM dorsomorphin (DM, A.G. Scientific), and 10 μM phenylhydrazonopyrazolone sulfonate 1 (PHPS1, Calbiochem, Darmstadt, Germany).

Inhibitor of SHP2 (10 μM PHPS1) was treated in NPCs for 7 days before neural differentiation and the inhibitor also added in cerebral organoids between 28 and 35 days of culture.

### Production of cerebral organoids from hiPSCs

Human cerebral organoids were generated from hiPSCs by using a protocol previously reported [43]. Briefly, hiPSC colonies were detached by treatment with 10 mg/ml collagenase IV (Invitrogen) for 5 min. Suspended hiPSC-clumps were placed on low-attachment dishes (SPL Life sciences) and then incubated in the hiPSC medium without FGF2 supplemented with 10 μM Y-27632 (ROCK inhibitor, AG scientific) for 24 h. For neural induction, suspended spheres were further cultured in the hiPSC medium supplemented with 10 μM dorsomorphin and 5 μM SB431542 for 5 days. cerebral organoids were incubated in the neural medium (NM) containing Neurobasal medium (Invitrogen), 1× B-27 supplement without vitamin A (Invitrogen), 1× Glutamax (Invitrogen), 20 ng/ml FGF2, and 20 ng/ml EGF for 19 days. The medium was changed daily for 10 days, and every other day for subsequent 9 days. For differentiation of neural cells from neural precursor cells, the NM medium with 20 ng/ml BDNF (Peprotech) and 20 ng/ml NT-3 (Peprotech) instead of FGF2 and EGF was used by day 25. For subsequent incubation, cerebral organoids were cultured in the neural medium (NM) containing Neurobasal medium (Invitrogen), 1× B-27 supplement without vitamin A (Invitrogen), and 1× Glutamax (Invitrogen) without using any bioreactors.

### Quantitative polymerase chain reaction (q-PCR)

Total RNA was isolated from the samples using an easy-Blue Total RNA Extraction kit (Intron Biotechnology, Inc.) according to the manufacturer’s protocol. Extracted RNA pellets were dissolved in diethylpyrocarbonate (DEPC)-treated distilled water (DW). Total RNA (1 μg) was reverse-transcribed using M-MLV Reverse transcriptase (Enzynomics, Daejeon, Korea) for 1 h. RT-PCR was performed with Taq Plus DNA polymerase (NanoHelix, Daejeon, Korea) on a GeneAmp® PCR system 9700 (Life Technologies). The PCR products were loaded on 2% agarose gel (EB agarose, LPS solution, Daejeon, Korea) containing red safe (Intron Biotechnology, Inc.). The bands of the PCR products were exposed under the UV lamp of a Gel 200 imaging system (Kodak, Rochester, New York, USA). Quantitative PCR (qPCR) amplification was performed with a pre-made 2× mixture on a CFX Connect™ Real-Time system (Bio-Rad, Hercules, CA, USA). The 2× mixture consisted of 40 mM Tris pH 8.4, 0.1 M KCl, 6 mM MgCl2, 2 mM dNTP, 0.2% fluorescein, 0.4% SYBR Green, and 10% DMSO. The primers used in this study are listed in Additional file 1 (Supplemental Table 1). The reaction conditions for the real-time q-PCR analysis were as follows: 95 °C for 5 min; 40 cycles of 95 °C for 10 s, 60 °C for 10 s, and 72 °C for 10 s; and then a melting curve. For the comparative analyses, the mRNA expression level of the genes was normalized to that of *GAPDH* and then expressed as a fold change relative to the expression level of the control. The ΔCt value was calculated as the difference between the GAPDH Ct and the target Ct. Fold changes in gene expression levels between the sample and control were determined using the formula 2^-(SΔCt−CΔCt)^.

### Immunofluorescence analysis

Cells were rinsed in phosphate-buffered saline (PBS, pH 7.4, Invitrogen) and fixed with 4% formalin solution (Sigma-Aldrich) for 30 min at room temperature (RT). After rinsing 3 times with PBS containing 0.1% Tween 20 (PBS-T, Sigma-Aldrich), the cells were treated with 0.1% Triton X-100 (MP Biomedicals, LLC, Santa Ana, California, USA) for 30 min at RT. The permeabilized cells were blocked with PBS containing 3% bovine serum albumin (BSA, Sigma-Aldrich). Cerebral organoids were harvested and fixed in 4% paraformaldehyde (PFA) for 24 h. Fixed cells were washed in PBS and cryoprotected in 30% buffered sucrose (Sigma Aldrich) at 4 °C for 72 h. Then, the organoids were made into gelatin-embedded block (7.5% gelatin in 10% sucrose/PBS) stored at − 80 °C. The organoids were sliced with 20 μm thick using a Cryostat (Leica microsystems, Wetzlar, Germany) and then moved on glass slides. The sections were blocked in PBS containing 3% BSA and 0.5% Triton X-100 for 2 h at RT. The primary antibodies and dilution ratios used in this study are listed in Additional file 1 (Supplemental Table 2). Cells were incubated with the primary antibody at 4 °C overnight. After washing with PBS-T, the samples were treated with each secondary antibody for 1 ~ 2 h. Secondary antibodies included Alexa 594-conjugated donkey anti-rabbit IgG (1:400, Invitrogen), Alexa 488-conjugated donkey anti-mouse IgG (1:400, Invitrogen), and Alexa 594-conjugated donkey anti-goat IgG (1:400, Invitrogen). After rinsing in PBS-T, the samples were counter-stained with 1 μg/ml 4′,6-diamidino-2-phenylindole dihydrochloride (DAPI, Sigma-Aldrich) for 10 min to stain the nuclei. Immuno-stained images were observed on a fluorescence microscope (Olympus) or a Zeiss LSM 780 confocal microscope equipped with argon and helium–neon lasers (Carl Zeiss, Oberkochen, Germany). The relative intensities of the fluorescence images were measured by ImageJ software (NIH, https://imagej.nih.gov/ij/). Dendritic and axonal lengths were traced and measured in neuronal cells by using the NeuronJ plugin of ImageJ software, respectively.

### Western blotting

Cells were harvested using RIPA buffer (Genetbio, Inc., Deajoen, Korea) containing protease inhibitor cocktails (Roche, Basel, Switzerland), 200 μM NaVO_3_, 100 mM NaF, and 200 μM PMSF on ice, transferred to a 1.5 ml tube, and homogenized by Vibra Cell™VCX-750 (Sonics & Materials, Inc., Newtown, Connecticut, USA). The protein concentration was determined using a microplate reader model 680 (Bio-Rad), and then 10 μg of the protein was mixed in 1× sample buffer (200 mM Tris-HCl pH 6.8, 4% SDS, 40% glycerol, 0.04% bromophenol blue, and 8% β-mercaptoethanol) and boiled on a hot plate for 5 min. The protein samples were separated on 10% SDS-polyacrylamide gel for 2.5 h and then transferred to nitrocellulose membranes (Whatman, New Jersey, USA) using a transfer tank (Hoefer, Inc., Holliston, MA, USA) for 3.5 h. The membrane was blocked with 4% skim milk (BD Biosciences) in Tris-buffered saline with Tween 20 (TBS-T, 100 mM Tris-HCl, 1.5 M NaCl, and 0.5% Tween 20) and incubated with the appropriate antibodies at 4 °C overnight. After rinsing with TBS-T, the membranes were incubated with horseradish peroxidase (HRP)-conjugated secondary antibody for 1 h at RT. Protein bands were illuminated on a LAS-4000 Mini (Fuji Film, Tokyo, Japan), and their intensities were measured using the ImageJ program. The measured values of phosphorylated and total protein were normalized to the intensity of GAPDH. The relative band intensities of the experimental group were compared with the control group. Antibodies used in this study are listed in Additional file 1 (Supplemental Table 2).

### FACS analysis

NPCs were dissociated via treatment with Accutase (eBioscience) for 10–15 min. Cell pellets were resuspended in PBS containing 1% BSA as FACS buffer and passed through the cap tube containing a cell strainer (BD Falcon) to filter the aggregated cell population. After centrifugation, the cells were incubated with PSA-NCAM (MAB5324, Millipore, RRID: AB_95211) antibody for 15 min at 4 °C. After washing with FACS buffer 3 times, the cells were incubated with Alexa Fluor® 488 goat-anti mouse IgM (μ chain, Invitrogen) as the secondary antibody for 10 min at 4 °C. After intensively rinsing with FACS buffer at least 5 times, the cells were fixed with 10% formalin solution (Sigma-Aldrich) and analyzed using a FACS Calibur flow cytometer (BD Biosciences). The PSA-NCAM-positive population was evaluated with FlowJo software (Tree Star, Ashland, OR, USA).

Neural cells were dissociated by trypsin/EDTA (GIBCO) for 5 min. Cerebral organoids were dissociated by treatment with Accutase (eBioscience) for 15 min. Dissociated neural cells and cerebral organoids were suspended in PBS containing 1% FBS and passed through cap tube involved in cell strainer (BD Falcon) to filter aggregated cells. After centrifugation, the cells were incubated with phycoerythrin (PE)-conjugated CD44 (550989, BD biosciences, RRID: AB_394000) antibody as a representative marker of glial cells [22, 51] for 30 min at 4 °C. After washing with FACS buffer twice, cells were fixed with 10% formalin solution (Sigma-Aldrich) and analyzed using a FACS Calibur flow cytometer (BD Biosciences). The CD44-positive population was analyzed using FlowJo software (Tree Star) OR, USA).

### Multi-electrode array

Differentiated neural cells were detached from the culture dishes by treatment with trypsin/EDTA for 5–10 min and dissociated by gentle pipetting. Enzyme activity was inactivated in neurobasal medium containing 10% FBS, 1× N2 and 0.5× B-27 supplement (Invitrogen), and 50 mM L-glutamine (Sigma-Aldrich). After centrifugation, the pellets were resuspended in 1 ml of the neurobasal medium. The neural cells were placed on electrodes of PLO/Laminin-coated MED probes (MED-P515A or R515A, Alpha MED Scientific Inc., Osaka, Japan) at a concentration of 100,000 cells/cm^2^ and cultured for 2 weeks before measuring the spontaneous spikes. The spontaneous spikes of neural cells were recorded on a MED64 system (Alpha MED Scientific, Inc.) under the neural medium for 5 min at 37 °C, 5% CO_2_ in air. Noise was filtered at 100 Hz using the Mobius program (Alpha MED Scientific, Inc.). Reliable spikes were tightly selected by setting a threshold of “6δ” and extracted in a time-dependent manner by a “spike sorting” analysis. Additionally, spikes representing positive waveforms were sorted from the extracted spikes. Raster plots were visualized to represent active channels using the MATLAB R2014a program (MathWorks, Inc., Natick, MA, USA). Active channels having more than 30 spikes were selected to analyze the number of extracellular spikes. Each spike frequency (Hz) equals to the number of extracellular spikes divided by the number of active channels in 300 s.

MEA recording of cerebral organoids was conducted using the Maestro Edge system (Axion BioSystems, Atlanta, GA, USA). A 24-well MEA plate containing a 16-electrode array per well was precoated with 0.05% polyethlenimine (PEI, Sigma Aldrich) and subsequently coated with 15 μg/ml PLO and 2 μg/ml laminin for overnight. Cerebral organoids were dissociated using Accutase for 15 min and placed on MEA plate at a density of 100,000 cells. Those cells were matured further 3 to 4 weeks. Spontaneous neural activities of cerebral organoids were measured for 10 min at an interval of 30 min at 37 °C, 5% CO_2_ in NM. Data acquisition and spontaneous spike recording were managed by using Axion Integrated Studio (AxIS 2.1). False-positive spikes and missed detection were minimized with setting a threshold of 6× SD. The Neural Metric Tool (Axion BioSystems) was used to explore the raster plots. The spike count files generated from the recordings were used to work out the number of spikes and spike frequency.

### Statistical analysis

The *N* value indicates independent experiments as biological replicates. All experiments performed with independently differentiated cells from WT-and NS-iPSCs. Most of the data are presented as the mean ± standard error of the mean (SEM). NS cell lines were compared with a WT-cell line. The *p* values were analyzed by Student’s *t* test using GraphPad Prism 5 (GraphPad Software, Inc., La Jolla, CA, USA): *p < 0.05, **p < 0.01, and ***p < 0.001.

## Results

### Noonan syndrome-iPSCs (NS-iPSCs) have pluripotent properties

Two NS patients (NS1 and NS2) had a point mutation (c.922A>G) in exon 8 of *PTPN11* on chromosome 12q24, which led to the replacement of asparagine (Asn) with aspartic acid (Asp) in the SHP2 protein. Another NS patient (NS3) had a point mutation (c.181G>A) in exon 3 of the same gene. Those NS patients were diagnosed with the typical phenotypes of Noonan syndrome, including facial dysmorphism, congenital heart defects, and developmental delay (Table 1).

**Table 1:** Information for patients with Noonan syndrome

Patient Information	Patient ID	**NS1**	**NS2**	**NS3**
	Age at biopsy	8 years	2.38 months (0.2 years)	1.2 months (0.1 years)
	Gender	Male	Female	Male
Genotype	Gene	PTPN11 (NC_000012.12)		
	Location	12q24.13		
	Nucleotide substitution	c.922A>G [**A**AT→**G**AT] in exon 8	c.922A>G [**A**AT→**G**AT] in exon 8	c.181G>A [**G**AT→**A**AT] in exon 3
	Amino acid substitution	p.Asn308Asp [N308D]	p.Asn308Asp [N308D]	p. Asp61Asn [D61N]
Symptoms	Facial dysmorphism	Typical face and short neck	Typical face, webbed and short neck	Typical face, webbed and short neck
	Stature	Short stature (− 2.7 SD score)	Short stature (− 3.35 SD score)	Short stature (− 1.93 SD score)
	Congenital heart defects	Pulmonary stenosis	Ventricular septal defect and trial septal defect	Pulmonary stenosis, atrial septal defect, and hypertrophic cardiomyopathy
	Neurological manifestations	Borderline mental retardation, learning disability and ADHD *Diagnosed at 8.1 years	Lower gross movement, social and language problem *Diagnosed at 36 months	Mild ADHD *Diagnosed at 5 years

*NS* Noonan syndrome, *PTPN11* protein tyrosine phosphatase, non-receptor type 11, *SD* standard deviation, *ADHD* attention deficit hyperactivity disorder

Three NS-iPSC lines generated from the patients were subjected to subsequent experiments. The NS-iPSCs expressed the pluripotent-associated markers (OCT4, SOX2, NANOG, TRA-1-60, and TRA-1-81) (Additional file 2: Fig. S1A). The NS-iPSCs were karyotypically normal (Additional file 2: Fig. S1B). A single point mutation was confirmed in the NS-Fib and NS-iPSCs by genomic sequencing (Additional file 2: Fig. S1C). Interestingly, basal p-ERK activity was slightly increased in the absence of basic fibroblast growth factor (bFGF), and the activities of p-SHP2 and p-ERK did not differ in the NS-iPSCs under bFGF treatment (10 and 20 min) compared with that of the WT-iPSCs (Additional file 3: Fig. S2). The NS-iPSCs exhibited pluripotency even with slight increments of the basal activity of p-ERK. Collectively, NS-iPSCs with SHP2 mutations normally maintained pluripotent characteristics.

### Activation of BMP and TGF-β signaling cause early neurodevelopmental impairment of NS-iPSCs

**Fig. 1:**
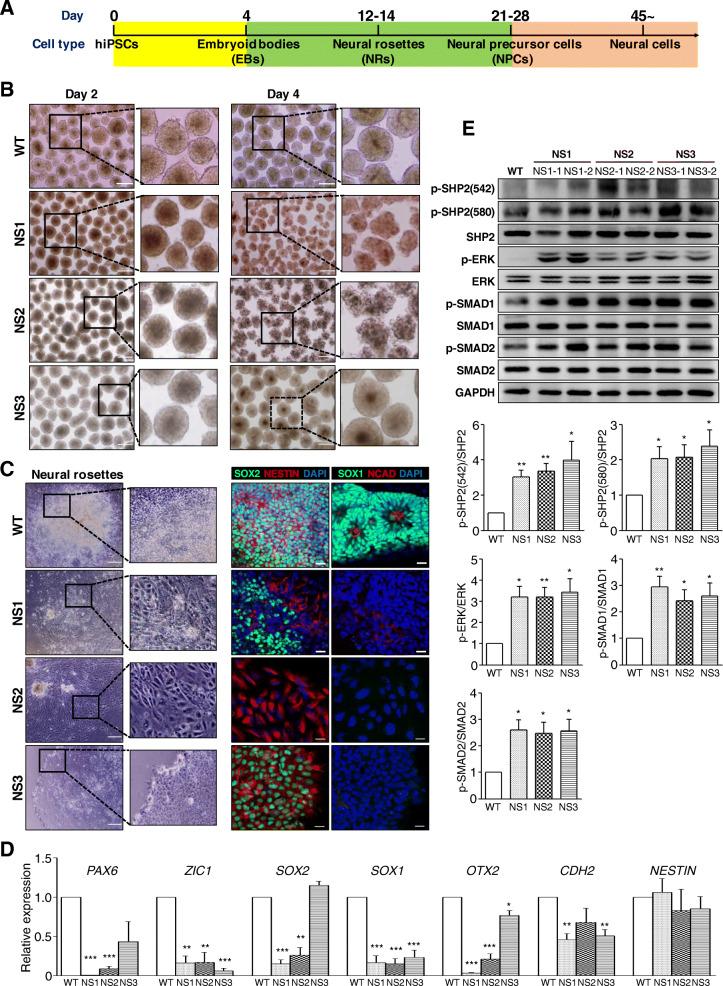
Defective early neural development of NS-iPSCs. **a** Schematic protocols for the neural differentiation of human iPSCs. **b** Aberrant maintenance of EBs derived from NS-iPSCs. NS-iPSCs normally formed EBs but showed impaired morphologies at day 4 during EB incubation, which was inconsistent with the WT-EBs. Scale bar, 200 μm. **c** Failure of NS-EBs to develop into NRs. NS-EBs failed to differentiate into neural rosettes. Expression of NR markers was abnormal in the NS derivatives. Blue color indicates nuclei stained with DAPI. Scale bars, 200 μm (bright field images) and 20 μm (fluorescence images). **d** Decreased transcription of neuroectodermal genes in the NS derivatives. Transcriptional level of neuroectodermal genes (*PAX6*, *ZIC1*, *SOX2, SOX*, *OTX2*, and *CDH2*) were significantly reduced in the NS derivatives. The relative expression levels are presented as the mean ± SEM (*n* = 3). **e** Increased activity of p-SHP2, p-ERK, p-SMAD1, and p-SMAD2 in the NS-EBs. The relative band intensities are presented as the mean ± SEM (*n* = 3). *p* values were determined using an unpaired Student’s *t* test. *p < 0.05, **p < 0.01, ***p < 0.001. Abbreviation: WT, wild-type; NS, Noonan syndrome; hiPSCs, human induced pluripotent stem cells

**Fig. 2:**
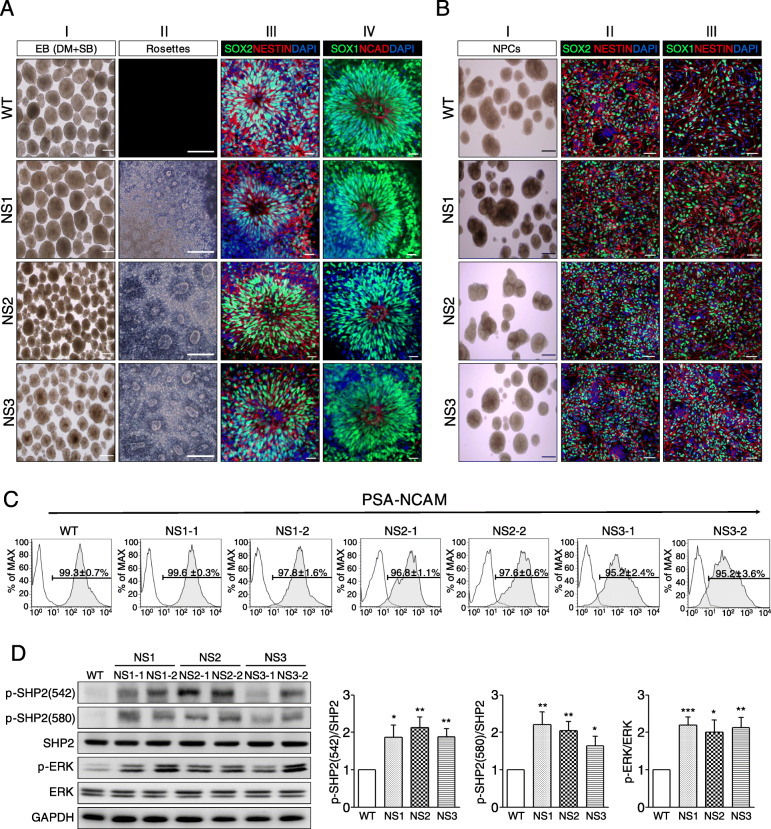
Effects of inhibited BMP and TGF-β pathways on EB formation in the NS-iPSCs. **a** Morphological recovery and NR develoment of NS-EBs by inhibition of both the BMP and TGF-β signaling pathways. Abnormal NS-EBs were ameliorated by treatment of BMP and TGF-β inhibitors (panel I). Similar to the WT-NRs, the NS-NRs had normal rosette structures (panel II) and expressed NR markers (panels III and IV). Scale bars, 100 μm (bright field images) and 20 μm (fluorescence images). **b** Normal development of NS-NRs to NPCs. Similar to the WT-NPCs, the NS-NPCs showed normal morphologies (panel I) and expressed neuroectodermal markers, such as SOX1, SOX2, and NESTIN (panel II and III). Scale bars, 500 μm (bright field images) and 50 μm (fluorescence images). **c** Immunofluorescence analyses of NS-NPCs with an immature neural marker, PSA-NCAM. The WT-NPCs and NS-NPCs showed a high proportion (more than 95%, *n* = 3) of PSA-NCAM positives (bottom). Black and tinted gray lines indicate the isotype control and PSA-NCAM, respectively. **d** Increased levels of p-SHP2 and p-ERK in the NS-NPCs. Western blot analysis showed that the levels of p-SHP2 and p-ERK were increased in the NS-NPCs compared with the WT-NPCs. The relative band intensities are presented as the mean ± SEM (*n* = 5). *p* values were determined using an unpaired Student’s *t* test. *p < 0.05, **p < 0.01, ***p < 0.001. Abbreviation: WT, wild-type; NS, Noonan syndrome; DM, dorsomorphin; SB, SB431542; PSA-CAM, polysialic-acid neural cell adhesion molecule

NS patients represent a range of defective cognitive functions [25, 52]. To explore whether SHP2 mutations are associated with aberrant neural development, NS-iPSCs differentiated into the neural cells by a stepwise method for cell specification, including EBs, NRs, NPCs, and neural cells (Fig. 1a). Embryoid bodies (EBs) developed from respective NS-iPSC lines were maintained for 2 days (Fig. 1b, left), but NS1- and NS2-EBs except NS3-EBs did not further develop by day 4 and showed jagged morphologies in appearance (Fig. 2b, right). Unlike WT-EBs, all NS-EBs failed to develop to neural rosettes (NRs) in which showed flattened morphologies without the columnar neuro-epithelial structures (Fig. 1c, left). Also, expression patterns of neuroectodermal markers (SOX2, NESTIN, SOX1, and NCAD) were abnormal in all NS-NRs (Fig. 1c, right). In addition, transcriptional levels of almost NR-associated genes (*PAX6*, *ZIC1*, *SOX2*, *SOX1*, *OTX2*, *CDH2*, and *NESTIN*) showed a lower in NS-NRs than WT-NRs (Fig. 1d). Thus, all NS-iPSCs failed to develop to early neuroectodermal lineage. The next question was whether impaired early neuroectodermal development in NS-iPSCs is associated with the activity among RAS-MAPK, BMP, and TGF-β signaling pathways. The levels of p-SHP2, p-ERK, p-SMAD1, and p-SMAD2 significantly increased in the NS-EBs compared with the WT-EBs (Fig. 1e). This result demonstrated that RAS-MAPK, BMP, and TGF-β signalings were activated in the NS-iPSCs during EB development. Our results indicate that aberrant activation of RAS-MAPK signaling as well as BMP signaling and TGF-β signaling may lead to impaired development of NS-iPSCs to the early neuroectodermal lineage.

### Inhibition of both BMP and TGF-β signalings induces the normal neuroectodermal development in NS-iPSCs

In the next experiment, we investigated whether the inhibition of the BMP and TGF-β signaling pathways ameliorates early neuroectodermal development in NS-iPSCs. Intriguingly, NS-EBs were morphologically rescued by both treatment with inhibitors of the BMP and TGF-β signaling pathways (Fig. 2a, panel I). Downregulation of p-ERK, p-SMAD1 and p-SMAD2 except p-SHP2 was observed in the chemicals-treated NS-EBs (Additional file 4: Fig. S3). Furthermore, the chemicals-treated EBs normally developed into NRs that express neuroectodermal markers, such as SOX2, NESTIN, SOX1, and NCAD (Fig. 2a, panels II–IV). However, inhibition of either the BMP or the TGF-β signaling pathway represented morphologically incomplete recovery of NS-EBs (Additional file 5: Fig. S4A). Suppression of the RAS-MAPK signaling pathway by treatment with SHP2 inhibitor was insufficient for the morphological development of NS-EBs (Additional file 5: Fig. S4B) and did not contribute to the development of neural rosettes (Additional file 5: Fig. S4C). Moreover, ERK inhibition by treatment of MEK inhibitor was not effective in appearances of NS-EBs (data not shown). These results suggest that the BMP and TGF-β signaling pathways, rather than the RAS-MAPK signaling pathway, play important roles in the early neuroectodermal development of NS-iPSCs.

In subsequent experiments, NS-NRs derived from the chemicals-treated NS-EBs differentiated into NPCs to test the subsequent developmental competence. NS-NPCs developed from NS-NRs had similar morphologies and sizes as the WT-NPCs (Fig. 2b, panel I). Attached NS-NPCs were morphologically normal and expressed neuroectodermal markers, such as SOX1, SOX2, and NESTIN (Fig. 2b, panels II and III). Similar to the WT-NPCs, the NS-NPCs differentiated from NS-NRs exhibited positive signals for polysialic-acid neural cell adhesion molecule (PSA-NCAM, an immature neural marker) (Fig. 2c). Although NS-NRs normally differentiated to the NPCs in appearance, the levels of p-SHP2 and p-ERK significantly increased in the NS-NPCs compared with the WT-NPCs (Fig. 2d). These results imply that activation of the ERK signaling pathway does not influence the development of NS-NRs to neural precursor cells.

### NS-neural cells represent enhanced gliogenesis and shortened neurites

**Fig. 3:**
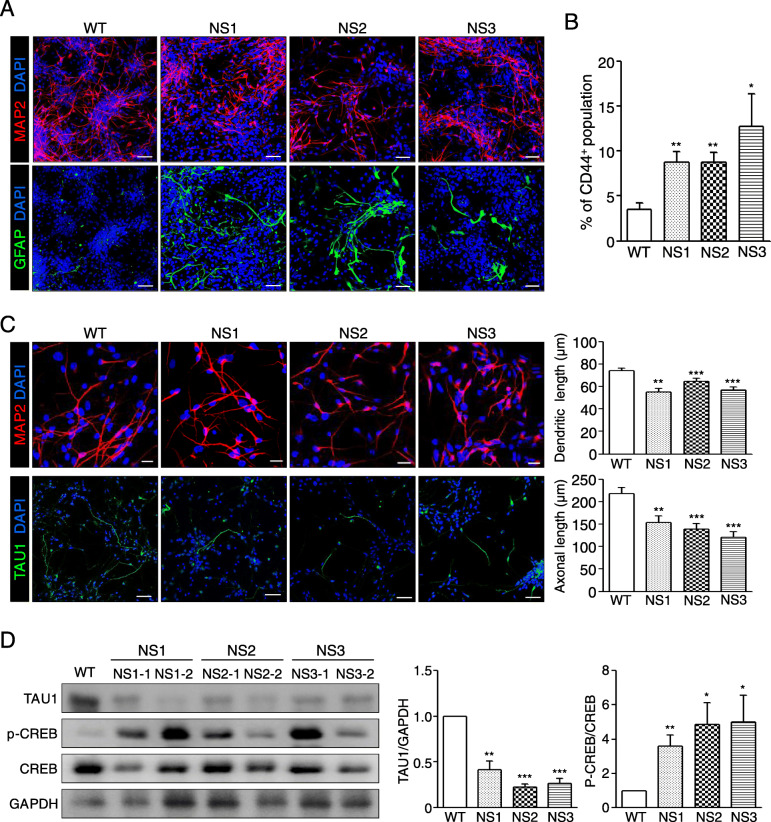
Defective differentiation of NS-NPCs into neural cells. **a** Aberrant gliogenesis of NS-neural cells. NS-NPCs were differentiated into neural cells under spontaneous conditions for 21–23 days. NS-neural cells showed a higher proportion of GFAP-positive cells compared with the WT-neural cells. MAP2 positive (red) and GFAP positive (green) represent neuronal and glial cells, respectively. Scale bar, 50 μm. **b** Increase of CD 44^+^ population in the NS-neural cells. The CD 44^+^ population in the NS-neural cells was analyzed by FACS. The percent of CD 44^+^ population are presented as the mean ± SEM (*n* = 4). **c** Reduced neurites of neuronal cells in the NS-neural cells. In neural cells, dendritic and axonal regions were detected by the MAP2 antibody (red) and TAU1 antibody (green), respectively. Lengths of the dendrites (*n* = 50 cells) and axons (*n* = 30 cells) were traced and measured using the ImageJ program. These comparisons were analyzed against the results of three independent experiments. Scale bars, 20 μm (MAP2) and 50 μm (TAU1). **d** Reduced expression of TAU1 and increments of p-CREB in the NS-neural cells. The relative band intensities are presented as the mean ± SEM (*n* = 4). *p* values were determined using an unpaired Student’s *t* test. *p < 0.05, **p < 0.01, ***p < 0.001. Abbreviation: WT, wild-type; NS, Noonan syndrome

Then, NS-NPCs further differentiated into neural cells in the absence of bFGF for 21–23 days. To test whether NS-NPCs have developmental potential towards neuronal and glial cells, NS-neural cells were immunostained with MAP2 and glial fibrillary acidic protein (GFAP). In the differentiation of the NPCs, the proportion of GFAP-positive cells in NS-neural cells was significantly higher than that of the WT-neural cells, whereas no difference was detected in the proportion of MAP2-positive cells between the WT- and NS-neural cells (Fig. 3a). In addition, proportion of GFAP-positive cells was significantly enhanced in NS-neural cells by FACS analysis using surface protein CD44 compared to WT-neural cells (Fig. 3b). Thus, differentiation of NS-NPCs to the neural cells resulted in a biased imbalance towards the glial cells.

Next, we questioned whether neuronal cells differentiated from NS-NPCs were normal or abnormal. Intriguingly, the NS-neural cells had shorter dendritic and axonal lengths than the WT-neural cells (Fig. 3c). It is reported that knockdown of tau in cortical pyramidal neuron derived from developing embryonic brains (E14.5–18) leads to reduction of dendritic complexity and extension [53]. In fact, expression of TAU1 was decreased in the NS-neural cells compared with the WT-neural cells (Fig. 3d). It is known that increment of phosphorylated cAMP-response element (CRE)-binding protein (CREB) decreases transcriptional expression of the tau in the brain of Alzheimer disease mice [54]. Levels of p-CREB were significantly enhanced in NS-neural cells compared with the WT-neural cells (Fig. 3d). Thus, it is likely that shortened dendritic and axonal lengths in the NS-neural cells are responsible for the reduction of TAU1 via activation of p-CREB. Collectively, these results represent that SHP2 mutations lead to abundancy of glial cells and shortened neurites of neuronal cells in NS-NPCs during the neural development in vitro.

### SHP2 inhibition rescues impairments of NS-neural cells

**Fig. 4:**
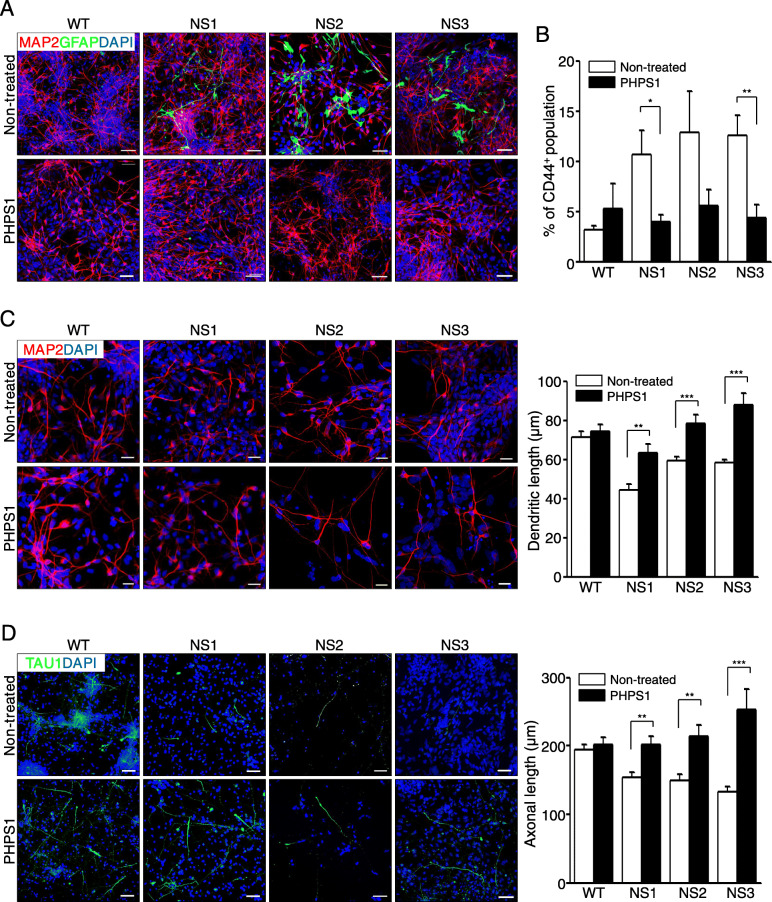
Recovery of abnormal phenotypes in the NS-neural cells by inhibition of SHP2 signaling. **a** Effects of SHP2 inhibition on gliogenesis in the NS-neural cells. WT-NPCs and NS-NPCs were treated with a SHP2 inhibitor (10 μM PHPS1) for 1 week and then differentiated into neural cells for 21–23 days. NS-neural cells treated with the inhibitor exhibited a lower proportion of GFAP-positive cells compared with the non-treated NS-neural cells. Scale bars, 50 μm. **b** Reduction of CD44^+^ population by SHP2 inhibition in the NS-neural cells. Percentages of CD 44^+^ population are expressed as the mean ± SEM (*n* = 3). **c** Extension of dendritic lengths in the NS-neural cells by SHP2 inhibition. Dendritic lengths were measured in the MAP2-positive cells of the WT- and NS-neural cells after treatment with the appropriate chemicals (*n* = 30 cells). The comparisons were analyzed against the results of three independent experiments. Scale bar, 20 μm. **d** Extension of axonal lengths in the NS-neural cells by SHP2 inhibition. Axonal lengths were measured in the TAU1-positive cells of the WT- and NS-neural cells after treatment with the appropriate chemicals (*n* = 30 cells). The comparisons were analyzed against the results from three independent experiments. Scale bar, 50 μm. *p* values were determined using an unpaired Student’s *t* test. *p < 0.05, **p < 0.01, ***p < 0.001. Abbreviation: WT, wild-type; NS, Noonan syndrome; PHPS1, phenylhydrazonopyrazolone sulfonate 1

NS-neural cells exhibited biased differentiation towards glial cells and shortened neurites (Fig. 3). Herein we thought that those anomalies might result from enhanced activities of SHP2 and ERK during the neural differentiation of NS-NPCs. We first investigated whether inhibition of SHP2 signaling influences the cell fate in the differentiation of NS-NPCs. Inhibition of SHP2 signaling by treatment with PHPS1 (SHP2 inhibitor) significantly decreased intensity of GFAP-positive signals and increased MAP2-positive signals in NS-neural cells (Fig. 4a). FACS analysis also represented reduction of CD44^+^ cells in NS-neural cells following the SHP2 inhibition (Fig. 4b). Secondly, we examined whether inhibition of SHP2 is associated with the development of neurites in NS-neural cells. As results, shortened dendritic and axonal lengths were significantly recovered to the normal range in NS-neural cells by inhibition of SHP2 signaling during in vitro differentiation, respectively (Fig. 4c, d). In addition, SHP2 inhibition enhanced the level of TAU1 expression and slightly normalized the activity of p-CREB in NS-neural cells (Additional file 6: Fig. S5A and S5B). Taken together, our findings suggest that SHP2 mutation is associated with imbalanced gliogenesis and defective development of neurites in NS-NPCs during neural differentiation.

### NS-cerebral organoids also show precocious gliogenesis and reduced neural activities

**Fig. 5:**
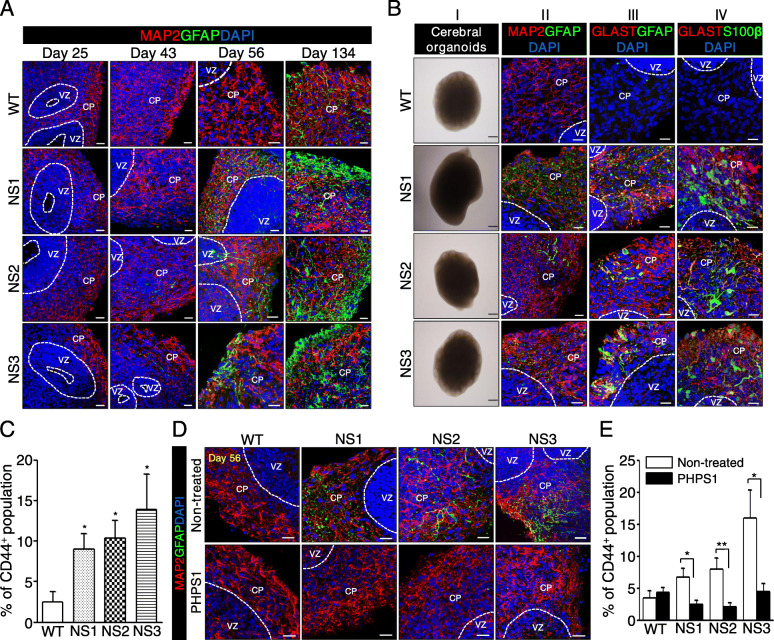
Increased gliogenesis in NS-cerebral organoids. **a** Time-dependent gliogenesis of NS-cerebral organoids. Scale bar, 20 μm. **b** Increment of glial cells in NS-cerebral organoids. NS-cerebral organoids expressed several glial cell markers such as GFAP (panel II), GLAST (panel III), and S100β (panel IV) in cortical plate zone. MAP2 expression was analogous between WT- and NS-cerebral organoids. Scale bar, 20 μm. **c** Increase of CD44 ^+^ population in the NS-cerebral organoids. Percentages of CD 44^+^ population are represented as the mean ± SEM (*n* = 5). **d** Reduction of GFAP-positive cells by SHP2 inhibition in NS-cerebral organoids. NS-cerebral organoids were transiently incubated with a SHP2 inhibitor (10 μM PHPS1) for 7 days (28–35th day), and then further differentiated for 3 weeks. NS-cerebral organoids treated with the inhibitor exhibited reduction of GFAP-positive cells compared with the non-treated NS-cerebral organoids. Scale bar, 20 μm. **e** Decreased proportion of CD44^+^ cells by SHP2 inhibition in NS-cerebral organoids. Percentages of CD44^+^ population are indicated as the mean ± SEM (*n* = 7). *p* values were determined using an unpaired Student’s *t* test. *p < 0.05, **p < 0.01. Abbreviation: WT, wild-type; NS, Noonan syndrome; VZ, ventricular zone; CP, cortical plate; PHPS1, phenylhydrazonopyrazolone sulfonate 1

Cerebral organoids are useful for studying spatiotemporal neurodevelopment of the human brain [45]. To know whether abnormal phenotypes in NS-neural cells are reproducible in 3D structures during neural development, cerebral organoids were produced from NS-iPSCs. Fig. S6A (Additional file 7) represents a protocol for the production of the cerebral organoids from human iPSCs. Like WT-iPSCs, NS-iPSCs normally developed to cerebral organoids that express neuroectodermal markers such as NESIN, SOX2, and SOX1 (Additional file 7: Fig. S6B), which is presumed as the neural progenitor stage, and further differentiated into neural cells expressing MAP2 (Additional file 7: Fig. S6C). To determine when gliogenesis appears during the development of NS-cerebral organoids, the organoids were collected at different days during in vitro culture. GFAP-positive cells were detected around 134 days of differentiation in WT-cerebral organoids, whereas they appeared from the 43rd day of differentiation in NS-cerebral organoids (Fig. 5a). The glial cells gradually increased in NS-cerebral organoids up to 134 days of differentiation. Thus, like 2D-cultured neural cells (Fig. 3a), NS-cerebral organoids showed precocious gliogenesis. Although NS-cerebral organoids cultured for 56 days had similar morphologies compared to WT ones, they also highly expressed diverse glial cell markers such as GFAP, GLAST, and S100β (Fig. 5b). FACS analysis displayed a higher proportion of CD44-positive cells in NS-organoids than WT ones (Fig. 5c). Thus, the fate of glial cells in NS-cerebral organoids seems to be determined early in the neurodevelopment process. Inhibition of SHP2 decreased populations of GFAP^+^ glial cells (Fig. 5d) and CD44^+^ cells in NS-cerebral organoids (Fig. 5e). Thus, SHP2 mutations lead to precocious appearance of astrocytes in NS-cerebral organoids during neural development in vitro.

**Fig. 6:**
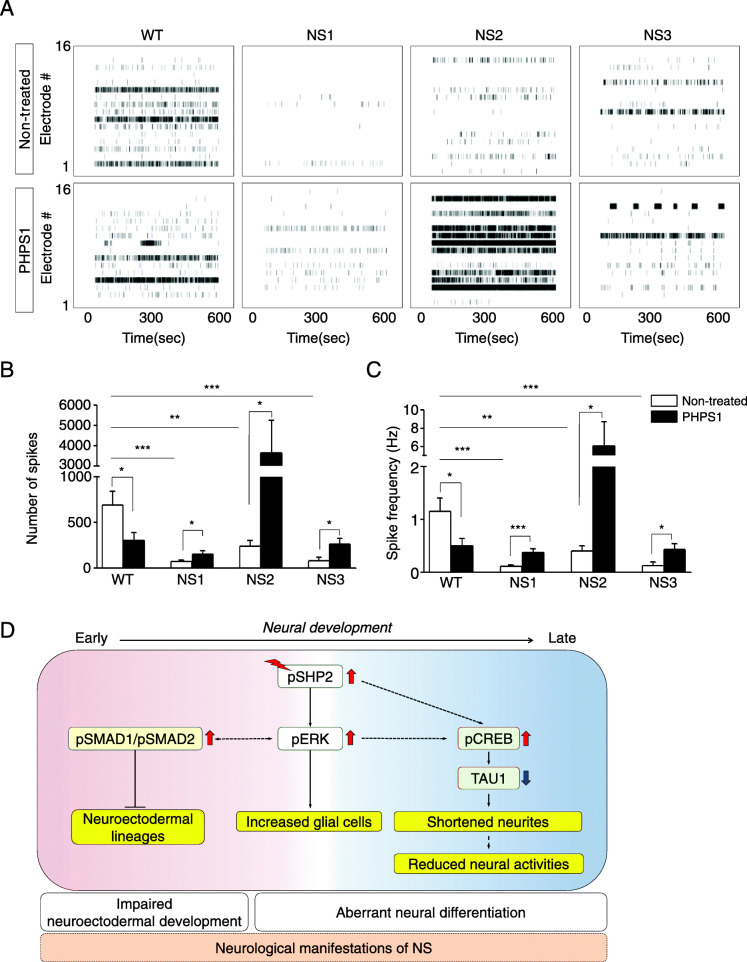
Extracellular spontaneous firing in NS-cerebral organoids. **a** Raster plot for extracellular spontaneous firing of cerebral organoids cultured for 62 day. **b**, **c** Number of extracellular spikes and spike frequency of cerebral organoids. Reduced number of extracellular spikes and spike frequency were improved by SHP2 inhibition in NS-cerebral organoids at day 62 of differentiation (*n* = 3). *p* values were determined using an unpaired Student’s *t* test. *p < 0.05, **p < 0.01, ***p < 0.001. **d** A putative model for neurodevelopmental abnormalities of NS-iPSCs. Abbreviation: WT, wild-type; NS, Noonan syndrome; PHPS1, phenylhydrazonopyrazolone sulfonate 1

To assess the functionality of NS-neural cells, the extracellular neural activity was examined in differentiated neural cells using a MEA system. The extracellular neural activity appeared to occur approximately 6 weeks after the induction of neural differentiation, and then the number of extracellular spikes gradually increased during culturing in a time-dependent manner in both the WT- and NS-neural cells during neural differentiation (Additional file 8: Fig. S7A). Interestingly, the extracellular neural activity was very low in the NS-neural cells at 12 weeks after the induction of neural differentiation (Additional file 8: Fig. S7B, upper panel). The total number of extracellular spikes reduced in NS-neural cells compared to WT-neural cells (Additional file 8: Fig. S7C, open bars). Treatment with SHP2 inhibitor partially rescued the extracellular neural activity in NS-neural cells (Additional file 8: Fig. S7B, lower panel, and S7C, filled bars). Nevertheless, spike frequency of the NS-neural cells was similar to those of the WT-neural cells (Additional file 8: Fig. S7D). The proportion of GFAP-positive cells decreased in the NS-neural cells at 12 weeks of neural differentiation after SHP2 inhibition (Additional file 8: Fig. S7E). To know whether the NS-cerebral organoids lead to low extracellular spontaneous activities, extracellular spontaneous activity was monitored between the 55th and 62th day during differentiation of cerebral organoids. No differences were observed in total number of spikes and spike frequency at 55 days of differentiation between control and SHP2 inhibition in NS-cerebral organoids (Additional file 9: Fig. S8). At 62 days of differentiation, intriguingly, NS-cerebral organoids showed low extracellular neural activity compared to WT ones (Fig. 6A, upper panel). SHP2 inhibition in NS-cerebral organoids improved the spontaneous firing (Fig. 6a, lower panel). The number of spikes and spike frequency were significantly increased in NS-cerebral organoids by SHP2 inhibition, respectively (Fig. 6b, c). These observations demonstrate that spontaneous neural activities decrease in both NS-neural cells and NS-cerebral organoids during neural development. Therefore, we suggest that SHP2 mutations cause dysfunctional electrophysiological property in NS-neural cells and NS-cerebral organoids.

## Discussion

Here we demonstrate for the first time that the neurological manifestations of NS may be responsible for the imbalance in neural development that leads to disproportionate numbers of neuronal and glial cells. In this study, two impairments were found in the neurodevelopmental process of NS-iPSCs during neural differentiation in vitro: defective maintenance of NS-EBs at the early stage and precocious gliogenesis in NS-neural cells and NS-cerebral organoids at the later stage. Various aberrant phenotypes, intriguingly, including enriched astrocytes, shortened neurites, and low electrophysiological activity was rescued in NS-neural cells and NS-cerebral organoids by SHP2 inhibition. Our results indicate that SHP2 mutation may contribute to imbalanced population of neurons and glial cells and reduced neurite outgrowth.

In this study, we used human iPSCs (hiPSCs) with the *PTPN11* mutations to model NS at the molecular and cellular levels during early neural development in vitro. SHP2 is ubiquitously expressed in the cytoplasmic region of cells [55] and regulates the self-renewal and differentiation in ESCs [56, 57]. Similar to WT-iPSCs, NS-iPSCs normally generated from dermal fibroblasts and maintained without any differentiation (Additional file 2: Fig. S1A). Interestingly, the level of p-SHP2 in the NS-iPSCs was similar to that of the WT-iPSCs (Additional file 3: Fig. S2). Thus, SHP2 mutations in NS-iPSCs may not affect the pluripotency competence. However, anomalies in the NS-iPSCs were observed in EB maintenance (Fig. 1b), and NS-EBs did not further develop into NRs (Fig. 1c). Similarly, CFC syndrome-iPSCs with the BRAF mutation show elevated SMAD1 signaling, which leads to failures of EB development and NR formation [40]. In the present study, the activities of p-SHP2, p-ERK, p-SMAD1, and p-SMAD2 were enhanced in the NS-EBs compared with the WT-EBs (Fig. 1e). Intriguingly, inhibition of both the BMP and TGF-β signaling pathways rescued EB development and subsequent NR formation in NS-iPSCs, whereas inhibition of p-SHP2 or p-ERK was not effective on rescuing EB and NR development (Additional file 5: Fig. S4B and S4C). Based on these results, the dual inhibition of the SMAD1 and SMAD2 signaling pathways is known to be effective for the neural induction of hESCs [58]. Elevated BMP signaling interrupts neural induction and results in induction towards the mesodermal lineage [59, 60]. Activin A treatment activates the expression of meso-endodermal genes in human and mouse ESCs [61]. These results indicate that both the BMP and TGF-β signaling pathways (but not the Ras-ERK signaling pathway) play important roles in the early neural induction of NS-iPSCs.

Although a variety of symptoms observed in NS patients are related to the activation of the RAS-MAPK pathway, it is unclear whether the SHP2 mutations influence cell fate determination during neural development. A SHP2 expression is detected in various brain tissues at the postnatal stage [62]. Here, we report that the SHP2 mutations are responsible for enhanced gliogenesis during neural development in humans. In this study, NS-NPCs preferentially developed to GFAP-positive glial cells even under neuronal induction conditions, whereas the WT-NPCs did not (Fig. 3a, b). *Ptpn11*^E76K/+^/*Nestin*-*Cre*^+^ conditional knock-in mice markedly increase the number of astrocytes in both cortex and hippocampus at 1 month old [63]. Ras-hyperactivating mutations, such as H-Ras G12V mutation and Nf1-inactivating mutation, cause an increase in gliogenesis in mice [64, 65]. Mek1/2 knockout results in the depletion of developmental gliogenesis in vivo [66]. NS-linked Raf1^L613V/wt^ mutant mice exhibit increased GFAP^+^ astrocytes and OLIG2^+^ oligodendrocyte progenitors in mature forebrain [67]. Therefore, it is likely that RAS-MAPK signaling is involved in the gliogenesis during neural development. Recently, it has been reported that strong expression of glial markers, including GFAP, S100β, and OLIG2, and elevated p-ERK levels are observed in the glioneuronal tumors or glioma of NS patients [26–28]. The levels of p-SHP2 and p-ERK increased in the NS-NPCs (Fig. 2d). Interestingly, SHP2 inhibition was effective on decreasing the numbers of GFAP-positive cells in the NS-neural cells and NS-cerebral organoids (Figs. 4a and 5d). Thus, elevated p-SHP2 activity seems to be associated with increment of gliogenesis in NS during neural development.

SHP2 plays a role in promoting neurogenesis by activating the Ras-Erk pathway and suppressing Jak-Stat3 signaling at embryonic days 13 and 14 during the progression of brain development in mice [22, 62]. Activation of the LIF-Jak-Stat3 pathway induces gliogenesis in the central nervous system [68]. In this study, we questioned whether increased glial development in the NS-NPCs might account for the activation of the JAK-STAT3 pathway. Although the NS-NPCs showed the same or low level of p-STAT3 compared with the WT-NPCs (Additional file 10: Fig. S9A), they retained their molecular and cellular properties (Fig. 2b, c). It is known that NOTCH signaling promotes transcriptional activation of GFAP in rodent cortical neural stem cells [69]. Sustained activation of LIF-STAT3 pathway enhances atrogliogenesis by activation of Smad 1 in mouse neuroepithelial cells [70]. In contrast, our results represent that JAK-STAT3, NOTCH, and BMP pathways do not seem to be critical for the cell fate determination of NS-NPCs towards glial cells (Additional file 10: Fig. S9A and S9B). Collectively, our results indicate that a gain-of-functional mutation of SHP2 gives rise to the precocious cell fate determination to gliogenesis during neural development.

In this study, another interesting finding was that the NS-neuronal cells had shortened dendrites and axons compared with the WT-neuronal cells. Similarly, neurons derived from CS-iPSCs (HRAS-G12V) exhibited shortened neurite length [37]. However, elevated Ras levels are known to be associated with increments of neurite outgrowth in neurons [71]. Nonetheless, the mechanisms underlying the induction of shortened dendritic and axonal lengths by the SHP2 mutation are unclear. The transcriptional expression of tau is suppressed by the activation of p-CREB [72]. In this study, NS-neural cells exhibited decreased TAU1 and increased p-CREB compared with that of the WT-neural cells (Fig. 3f). Nonetheless, it remains elusive whether the shortened lengths of dendrites and axons are associated with increased p-CREB via elevated p-ERK in the NS-neural cells. Midbrain dopaminergic neurons with the LRRK-G2019S mutation exhibit shortened neurite outgrowth by the activation of p-ERK [73]. Fragile X syndrome-derived neurons exhibit increased p-ERK as well as shortened neurite lengths [74]. Here, SHP2 inhibition led to an increase of TAU1 protein expression and a decrease of p-CREB activity in the NS-neural cells (Additional file 6: Fig. S5). Our results demonstrate that the SHP2 mutations cause defective neuronal development in the NS-neural cells through low TAU1 expression and p-CREB hyperactivation.

In this study, NS-neural cells exhibited unbalanced cell fate decisions into glial cells and shortened neural morphologies (Fig. 3). We questioned whether these anomalies in the NS-neural cells influence neural functions. As shown in Fig. 6 and S7 (Additional file 8), the NS-neural cells and NS-cerebral organoids exhibited a decrease in the number of extracellular spikes compared to WT ones. Therefore, reduction of extracellular spontaneous firing seems to be responsible for shortened neurites in NS-neural cells and NS-cerebral organoids. In addition, SHP2 inhibition not only extended neurite lengths in NS-neuronal cells (Fig. 4c, d), but also recovered extracellular neural activities in NS-neural cells and NS-cerebral organoids (Fig. 6 and S7). Nonetheless, it is still uncertain whether enhanced glial cells affect the extracellular neural activity in NS-neural cells and NS-cerebral organoids.

Based on our results, we propose a putative model for neurodevelopmental defects in NS-iPSCs during neural differentiation in vitro (Fig. 6d). SHP2 mutations excessively activated ERK, BMP, and TGF-β signaling pathways and then impaired early neuroectodermal development in NS-iPSCs. We found that both BMP and TGF-ß signaling pathways, not ERK signaling pathway, were critical for early neuroectodermal development in NS-iPSCs. Thereafter, various neurodevelopmental anomalies (increased glial cells, shortened neurites, and reduced extracellular neural activities) were observed in NS-neural cells and NS-cerebral organoids. Interestingly, SHP2 inhibition rescued those anomalies in NS-neural cells and NS-cerebral organoids. Taken together, we suggest that early and late combinatorial anomalies are associated with neurological impairments in Noonan syndrome.

## Conclusions

In vitro disease modeling for the neurological dysfunctions of NS patients has not been reported to date. In this study, we observed early neuroectodermal defects and imbalance between neuronal and glial cells in NS-iPSCs during neural development. Our results suggest possible deficiencies that can affect cognitive impairments during brain development in NS patients.

## Supplementary information

**Additional file 1: Table S1.** Primers^a^ used in this study **Table S2.** Primary antibodies used for the immunofluorescence and western blotting assays.

**Additional file 2: Figure S1.** Characterization of NS-iPSCs. (A) Expression of pluripotency markers in the NS-iPSCs. Similar to the WT-iPSCs, the NS iPSCs expressed various pluripotency markers, such as OCT4, SOX2, NANOG, TRA-1-60, and TRA-1-81. Scale bar, 200 μm. (B) Normal karyotypes of NS-iPSCs. (C) Single point mutation of the PTPN11 gene in the NS-iPSCs. The point mutation of the PTPN11 gene was verified by DNA sequencing.

**Additional file 3: Figure S2.** Activation of p-ERK upon bFGF stimulation in NS-iPSCs. WT-iPSCs and NS-iPSCs were starved in hPSC medium containing 0.1% SR without bFGF for 6 hr and then incubated in hPSC medium supplemented with 20 ng/ml bFGF for 10 and 20 min. Similar to the WT-iPSCs, the activity of p-ERK in the NS-iPSCs was slightly enhanced upon starvation. The relative band intensities are presented as the mean ± SEM (*n*=3). *P* values were determined by using an unpaired Student’s t-test. *, *p* < 0.05; **, *p* < 0.01; ***, *p* < 0.001.

**Additional file 4: Figure S3.** Downregulation of p-ERK, p-SMAD1, and p-SMAD2 by dual inhibition of BMP and TGF-β signaling in the NS-EBs. Dual inhibition downregulated the levels of p-ERK, p-SMAD1, and p-SMAD2 but did not affect the activity of p-SHP2 in the NS-EBs. The relative ratios are presented as the mean ± SEM (*n*=2).

**Additional file 5: Figure S4.** Treatments of NS-iPSCs with diverse chemicals during EB formation. (A) Effects of BMP inhibitor and TGF-β inhibitor on EB formation in NS-iPSCs. Treatment of either BMP inhibitor or TGF-β inhibitor alone was not effective for the morphological recovery of NS-EBs. NS-EBs were morphologically improved by the dual inhibition of BMP and TGF-β signaling. Scale bar, 200 μm. (B) Effects of SHP2 inhibition on EB formation in NS-iPSCs. NS-iPSCs were independently incubated with 10 μM PHPS1 (SHP2 inhibitor). SHP2 inhibition did not improve EB formation in NS-iPSCs. Scale bar, 200 μm. (C) Developmental failure of NS-EBs treated with SHP2 inhibitor to NR. Scale bar, 20 μm.

**Additional file 6: Figure S5.** Regulation of TAU1 and p-CREB level by SHP2 inhibition in NS-neural cells (A) Expression of TAU1 in the NS-neural cells by SHP2 inhibition. Protein level of TAU1 was slightly increased in PHPS1-treated NS-neural cells compared with the non-treated NS neural cells. The relative band intensities are presented as the mean ± SEM (*n*=3). (B) Protein level of p-CREB in the NS-neural cells by SHP2 inhibition. The relative band intensities are presented as the mean ± SEM (n=2). P values were determined by using an unpaired Student’s t-test. *, p < 0.05; **, *p* < 0.01.

**Additional file 7: Figure S6.** Characterization of cerebral organoids developed from human iPSCs. (A) Schematic protocol for differentiation of cerebral organoids from human iPSCs. (B) Expressions of neuroectodermal markers in cerebral organoids at 25 day of culture. Scale bars, 50 μm. (C) Expression of neuroectodermal and neuronal markers in cerebral organoids. WT-and NS-cerebral organoids retained neuroectodermal cells expressing SOX2 in ventricular zone (VZ) and neuronal cells expressing MAP2 in cortical plate (CP). Scale bars, 50 μm.

**Additional file 8: Figure S7.** Time-course extracellular neural activities of NS-neural cells. (A) Monitoring of number of spontaneous extracellular spikes during neural differentiation. In WT- and NS-neural cells, recording of extracellular neural activities were obtained for 5 min at a two-week interval from 6 to 12 weeks during neural differentiation from NPCs. The number of extracellular spikes was significantly reduced in NS-neural cells at 12 week. Data were represented as mean ± SEM (6 week, *n*=5; 8 week, *n*=3; 10 week, n=2; 12 week, n=2). (B) Raster plot of extracellular spikes of NS-neural cells cultured for 12 weeks. Active channels with extracellular spikes in NS-neural cells were fewer than WT-neural cells (non treated, upper panel). A number of active channels increased via SHP2 inhibition in NS neural cells (lower panel). Extracellular spikes are shown as dots among the 64 electrodes. The bin size is 1 ms. The electrodes with extracellular spikes are defined as active channels. (C) Comparison of number of extracellular spikes between non- and PHPS1-treated groups. In the non-treated group (open bar), a small number of extracellular spikes were detected in NS-neural cells compared to WT-neural cells. SHP2 inhibition partially increased number of extracellular spikes in NS-neural cells (filled bar). (D) Spike frequency of NS-neural cells. No difference was detected in the spike frequency of neural cells between non- and SHP2 inhibited groups. (E) Decrease of glial cells in NS-neural cells after SHP2 inhibition. Glial cells were reduced in NS-neural cells after treatment of PHPS1. MAP2-positive (red) and GFAP-positive (green) cells represented neuronal and glial cells, respectively. Scale bar, 50 μm. These results were repeated twice independently with a different set of neural cells. *P* values were determined by using an unpaired Student’s t-test. *, *p* < 0.05; **, *p* < 0.01.

**Additional file 9: Figure S8.** Spontaneous neural activities of cerebral organoids at 55 day of culture (A) Raster plots for extracellular spikes measured in cerebral organoids at 55 day of culture. (B-C) Number of spikes and spike frequency in cerebral organoids. These results were repeated from independently generated cerebral organoids (n = 3).

**Additional file 10: Figure S9.** Activities of STAT3, NOTCH and BMP signalings in NS NPCs (A) Activity of p-STAT3 in the NS-NPCs. Level of p-STAT3 in NS-NPC was lower than WT ones. (B) Levels of cleaved NOTCH and p-SMAD1 in NS-NPCs. There was no difference in the level of cleaved NOTCH and p-SMAD1 between WT- and NS-NPCs. The relative band intensities are presented as the mean ± SEM (*n*=4). P values were determined by using an unpaired Student’s t-test. **, p < 0.01; ***, *p* < 0.001.

## Data Availability

The datasets during and/or analyzed during the current study are available from the corresponding author on reasonable request.

## Abbreviations

NS: Noonan syndrome
PTPN11: Tyrosine-protein phosphatase non-receptor type 11
SHP2: Src homology 2 domain-containing protein tyrosine phosphatase 2
iPSCs: Induced pluripotent stem cells
EBs: Embryoid bodies
NRs: Neural rosettes
NPCs: Neural precursor cells
CS: Costello syndrome
CFC: Cardiofaciocutaneous
WT: Wild-type
TGF-β: Transforming growth factor-β
BMP: Bone morphogenetic protein
hiPSCs: Human induced pluripotent stem cells
ERK: Extracellular signal-regulated kinase
STAT3: Signal transducer and activator of transcription 3
VZ: Ventricular zone
CP: Cortical plate
bFGF: Basic fibroblast growth factor
FBS: Fetal bovine serum
PSA-NCAM: Polysialic-acid neural cell adhesion molecule
PHPS1: Phenylhydrazonopyrazolone sulfonate 1
MAP2: Microtubule-associated protein 2
GFAP: Glial fibrillary acidic protein
GLAST: Glutamate ASpartate Transporter 1
S100β: S100 calcium-binding protein B
SEM: Standard error of the mean
