# Correction to: SHP2 mutations induce precocious gliogenesis of Noonan syndrome-derived iPSCs during neural development in vitro

**DOI:** 10.1186/s13287-020-01775-8

**Published:** 2020-07-16

**Authors:** Younghee Ju, Jun Sung Park, Daejeong Kim, Bumsoo Kim, Jeong Ho Lee, Yoonkey Nam, Han-Wook Yoo, Beom Hee Lee, Yong-Mahn Han

**Affiliations:** 1 Department of Biological Sciences, KAIST, Daejeon, 34141 Republic of Korea; 2 Graduate School of Medical Science and Engineering, KAIST, Daejeon, 34141 Republic of Korea; 3 Department of Bio and Brain Engineering, KAIST, Daejeon, 34141 Republic of Korea; 4 Department of Pediatrics, Asan Medical Center Children’s Hospital, University of Ulsan College of Medicine, Seoul, 05505 Republic of Korea

**Correction to: Stem Cell Res Ther (2020) 11: 209**

**https://doi.org/10.1186/s13287-020-01709-4**

The original article [1] contained an error in Fig. 2 whereby the 2nd panel in the top row of Fig. 2A was mistakenly blanked during the production process by the team handling the manuscript. Fig. 2A in the original article has since been corrected.

## References

[1] Ju Y (2020). SHP2 mutations induce precocious gliogenesis of Noonan syndrome-derived iPSCs during neural development in vitro. Stem Cell Res Ther.

