# Supplementary material for: SHP2 mutations induce precocious gliogenesis of Noonan syndrome-derived iPSCs during neural development in vitro
Source: Stem Cell Res Ther. 2020 Jun 3;11:209. doi: 10.1186/s13287-020-01709-4 (PMC7268229; doi:10.1186/s13287-020-01709-4)
Supplement: Supplementary file 1 — Additional file 1: Table S1. Primersa used in this study Table S2. Primary antibodies used for the immunofluorescence and western blotting assays. [file 13287_2020_1709_MOESM1_ESM.pdf]

Additional file 1: Supplemental Tables

Supplemental Table 1. Primers[a] used in this study

Gene	Accession No	Forward	Reverse	Size
PAX6	NM_000280.4	GTGTCCAACGGATGTGTGAG	CTAGCCAGGTTGCGAAGAAC	254
ZIC1	NM_003412.4	GCGCTCCGAGAATTTAAAGA	CGTGGACCTTCATGTGTTTG	212
SOX2	NM_003106.4	ACTGGCGAACCATCTCTGTG	AATTACCAACGGTGTCAACCTG	118
SOX1	NM_005986.3	CCTCCGTCCATCCTCTG	AAAGCATCAAACAACCTCAAG	201
OTX2	NM_021728.4	GAAGCACTGTTTGCCAAGACC	CTCCATTCTGCTGTTGTTGCT	151
CDH2	NM_001792.5	GATATGCTTCAACACGCTTT	CCAAGATAATAAAATCGCTCCAT	98
NESTIN	NM_006617.2	GCAGGAGAAACAGGGCCTAC	AAAGCTGAGGGAAGTCTTGGA	175
GAPDH	NM_001289745	CTTCGCTCTCTGCTCCTCCT	GTTAAAAGCAGCCCTGGTGA	152

[a]Primers were able to detect all of the isoforms of each gene.

Supplemental Table 2. Primary antibodies used for the immunofluorescence and western blotting assays

Primary antibody	Species	Dilution	Company	Cat.no	AB Registry ID (RRID)
OCT4	Goat	1:200	Santacruz	sc-8628	AB_653551
SOX2	Rabbit	1:200	Cell signaling	#3579S	AB_2195767
NANOG	Rabbit	1:200	Cell signaling	#3580S	AB_2150399
TRA-1-60	Mouse	1:200	Milipore	MAB4360	AB_2119183
TRA-1-81	Mouse	1:200	Milipore	MAB4381	AB_177638
NESTIN	Mouse	1:100	Milipore	MAB5326	AB_2251134
SOX1	Goat	1:300	R&D systems	AF3369	AB_2239879
NCAD	Mouse	1:200	BD sciences	610920	AB_2077527
MAP2	Mouse	1:1000	Sigma-Aldrich	M1406	AB_477171
TAU1	Mouse	1:200	Milipore	MAB3420	AB_94855
GFAP	Rabbit	1:1000	Abcam	Ab7260	AB_305808
GLAST (ASCA-1)	Mouse	1:300	MACS Meltenyi Biotec	130-095-822	AB_10829302
S100β	Rabbit	1:300	Proteintech	15146-1-AP	AB_2254244
pY542-SHP2	Rabbit	1:500	Cell signaling	#9793S	AB_10694365
pY580-SHP2	Rabbit	1:500	Cell signaling	#9793S	AB_10694365
SHP2	Rabbit	1:500	Cell signaling	#9793S	AB_10694365
Phospho-p42/44 MAPK (p-ERK1/2)	Rabbit	1:1000	Cell signaling	#4370	AB_2315112
p42/44 MAPK (ERK1/2)	Rabbit	1:1000	Cell signaling	#9102	AB_330744
p-SMAD1	Rabbit	1:500	Cell signaling	#9511	AB_331671
SMAD1	Rabbit	1:500	Cell signaling	#9743	AB_2107780
p-SMAD2	Rabbit	1:500	Cell signaling	#3108	AB_490941
SMAD2	Rabbit	1:500	Cell signaling	#3102	AB_331675
p-STAT3	Mouse	1:500	Santacruz	sc-8059	AB_628292
STAT3	Mouse	1:500	Santacruz	Sc-8019	AB_628293
Cleaved Notch1(Val1744)	Rabbit	1:500	Cell signaling	#4147	AB_2153348
Notch1 (C-20)	Goat	1:500	Santacruz	sc-6014	AB_650336
p-CREB(Ser133)	Rabbit	1:1000	Cell signaling	9198S	AB_2561044
CREB	Mouse	1:1000	Cell signaling	9104S	AB_10691832
HRP-conjugated GAPDH	Rabbit	1:1000	Santacruz	Sc-25778	AB_10167668
