# Supplementary figures and images for: SHP2 mutations induce precocious gliogenesis of Noonan syndrome-derived iPSCs during neural development in vitro
Source: Stem Cell Res Ther. 2020 Jun 3;11:209. doi: 10.1186/s13287-020-01709-4 (PMC7268229; doi:10.1186/s13287-020-01709-4)

## Additional File 2: Fig. S1

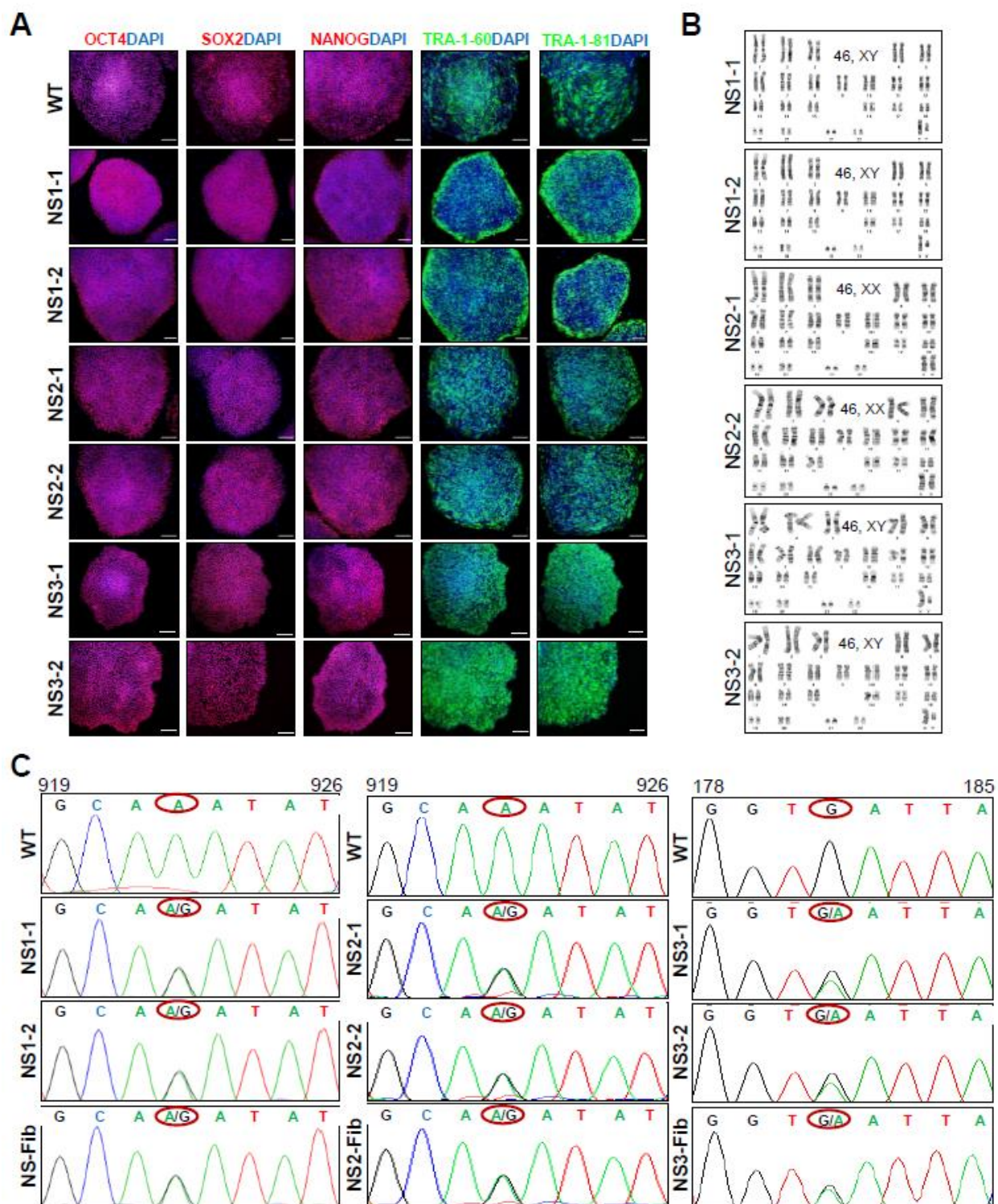

Supplement: Supplementary file 2 — Additional file 2: Figure S1. Characterization of NS-iPSCs. (A) Expression of pluripotency markers in the NS-iPSCs. Similar to the WT-iPSCs, the NS iPSCs expressed various pluripotency markers, such as OCT4, SOX2, NANOG, TRA-1-60, and TRA-1-81. Scale bar, 200 μm. (B) Normal karyotypes of NS-iPSCs. (C) Single point mutation of the PTPN11 gene in the NS-iPSCs. The point mutation of the PTPN11 gene was verified by DNA sequencing. [file 13287_2020_1709_MOESM2_ESM.pdf]

## Additional File 3: Fig. S2

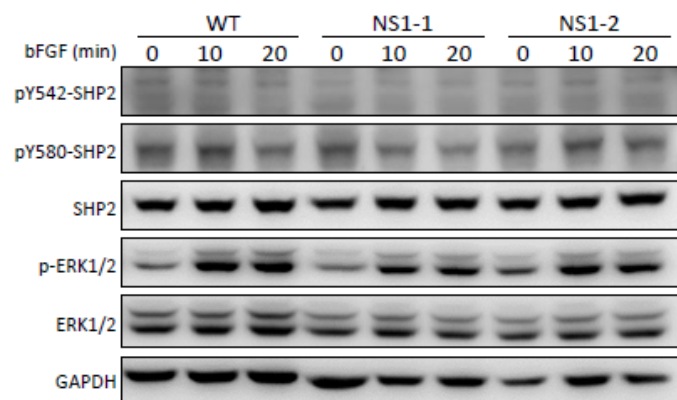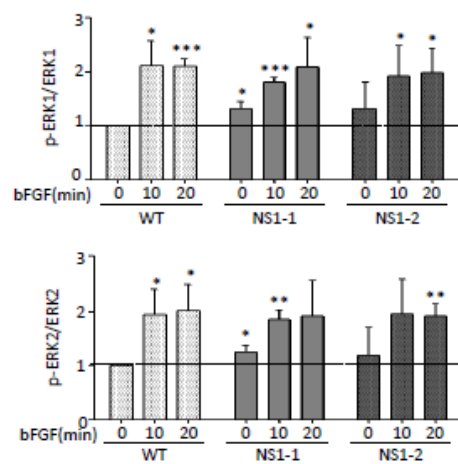

Supplement: Supplementary file 3 — Additional file 3: Figure S2. Activation of p-ERK upon bFGF stimulation in NS-iPSCs. WT-iPSCs and NS-iPSCs were starved in hPSC medium containing 0.1% SR without bFGF for 6 hr and then incubated in hPSC medium supplemented with 20 ng/ml bFGF for 10 and 20 min. Similar to the WT-iPSCs, the activity of p-ERK in the NS-iPSCs was slightly enhanced upon starvation. The relative band intensities are presented as the mean ± SEM (n=3). P values were determined by using an unpaired Student’s t-test. *, p < 0.05; **, p < 0.01; ***, p < 0.001. [file 13287_2020_1709_MOESM3_ESM.pdf]

**Additional File 4: Fig. S3**

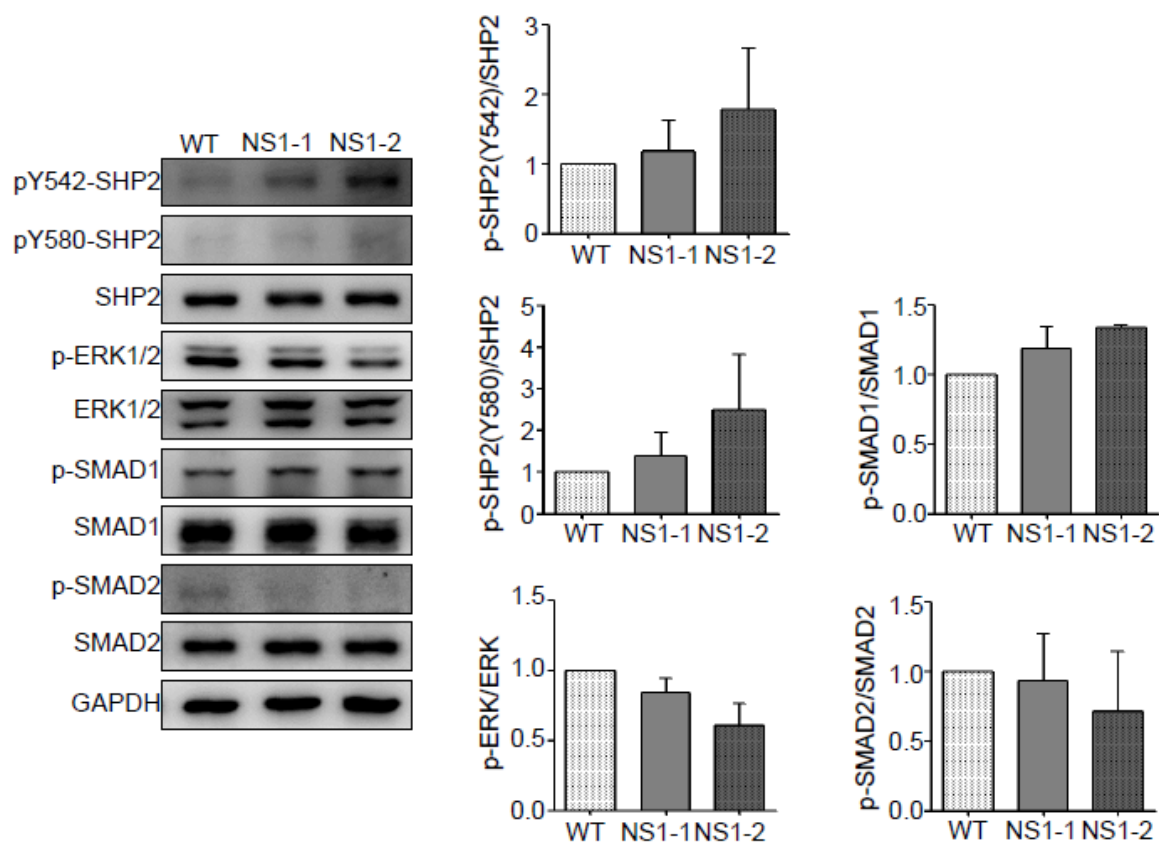

Supplement: Supplementary file 4 — Additional file 4: Figure S3. Downregulation of p-ERK, p-SMAD1, and p-SMAD2 by dual inhibition of BMP and TGF-β signaling in the NS-EBs. Dual inhibition downregulated the levels of p-ERK, p-SMAD1, and p-SMAD2 but did not affect the activity of p-SHP2 in the NS-EBs. The relative ratios are presented as the mean ± SEM (n=2). [file 13287_2020_1709_MOESM4_ESM.pdf]

## Additional File 5: Fig. S4

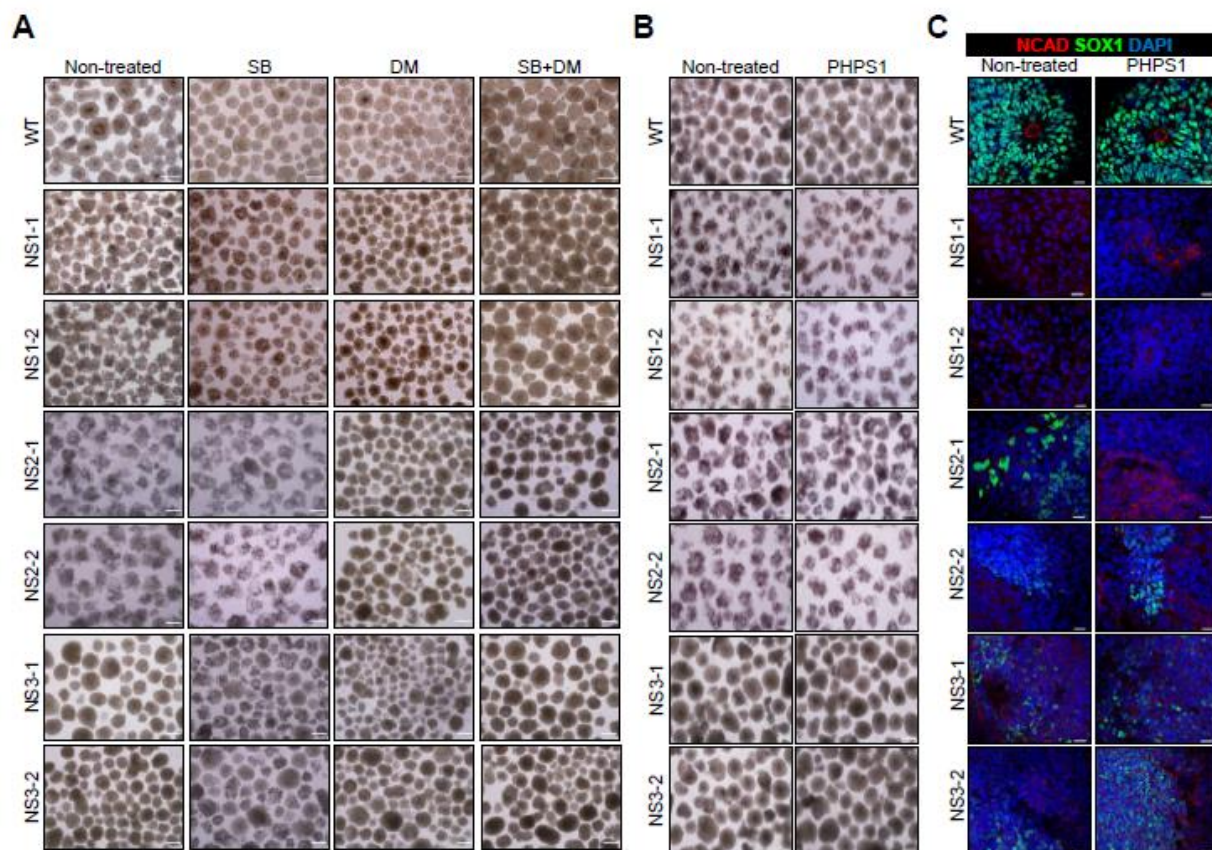

Supplement: Supplementary file 5 — Additional file 5: Figure S4. Treatments of NS-iPSCs with diverse chemicals during EB formation. (A) Effects of BMP inhibitor and TGF-β inhibitor on EB formation in NS-iPSCs. Treatment of either BMP inhibitor or TGF-β inhibitor alone was not effective for the morphological recovery of NS-EBs. NS-EBs were morphologically improved by the dual inhibition of BMP and TGF-β signaling. Scale bar, 200 μm. (B) Effects of SHP2 inhibition on EB formation in NS-iPSCs. NS-iPSCs were independently incubated with 10 μM PHPS1 (SHP2 inhibitor). SHP2 inhibition did not improve EB formation in NS-iPSCs. Scale bar, 200 μm. (C) Developmental failure of NS-EBs treated with SHP2 inhibitor to NR. Scale bar, 20 μm. [file 13287_2020_1709_MOESM5_ESM.pdf]

## Additional file 6: Fig. S5

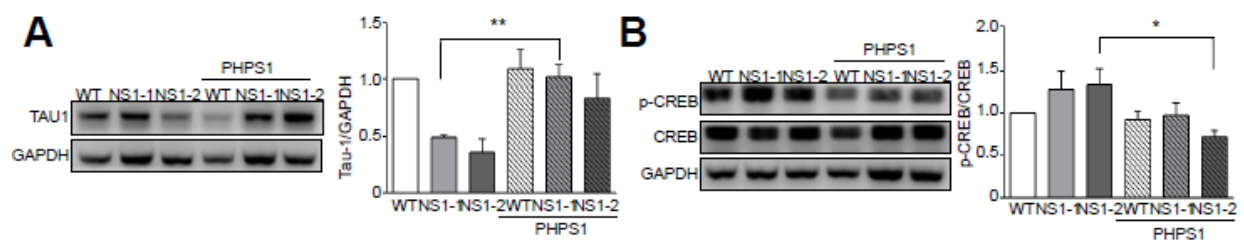

Supplement: Supplementary file 6 — Additional file 6: Figure S5. Regulation of TAU1 and p-CREB level by SHP2 inhibition in NS-neural cells (A) Expression of TAU1 in the NS-neural cells by SHP2 inhibition. Protein level of TAU1 was slightly increased in PHPS1-treated NS-neural cells compared with the non-treated NS neural cells. The relative band intensities are presented as the mean ± SEM (n=3). (B) Protein level of p-CREB in the NS-neural cells by SHP2 inhibition. The relative band intensities are presented as the mean ± SEM (n=2). P values were determined by using an unpaired Student’s t-test. *, p < 0.05; **, p < 0.01. [file 13287_2020_1709_MOESM6_ESM.pdf]

# Additional File 7: Fig. S6

**A**

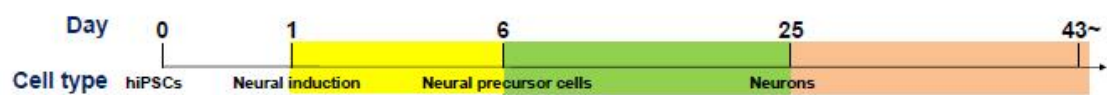

**B**

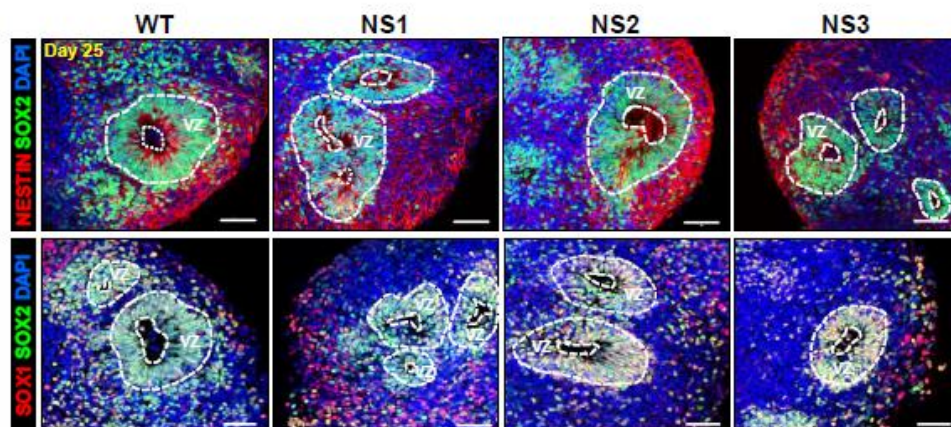

**C**

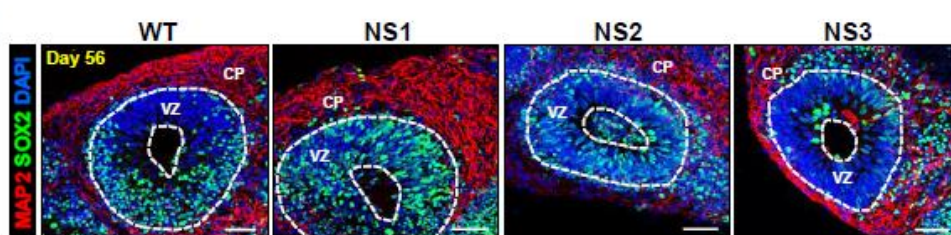

Supplement: Supplementary file 7 — Additional file 7: Figure S6. Characterization of cerebral organoids developed from human iPSCs. (A) Schematic protocol for differentiation of cerebral organoids from human iPSCs. (B) Expressions of neuroectodermal markers in cerebral organoids at 25 day of culture. Scale bars, 50 μm. (C) Expression of neuroectodermal and neuronal markers in cerebral organoids. WT-and NS-cerebral organoids retained neuroectodermal cells expressing SOX2 in ventricular zone (VZ) and neuronal cells expressing MAP2 in cortical plate (CP). Scale bars, 50 μm. [file 13287_2020_1709_MOESM7_ESM.pdf]

## Additional file 8: Figure S7

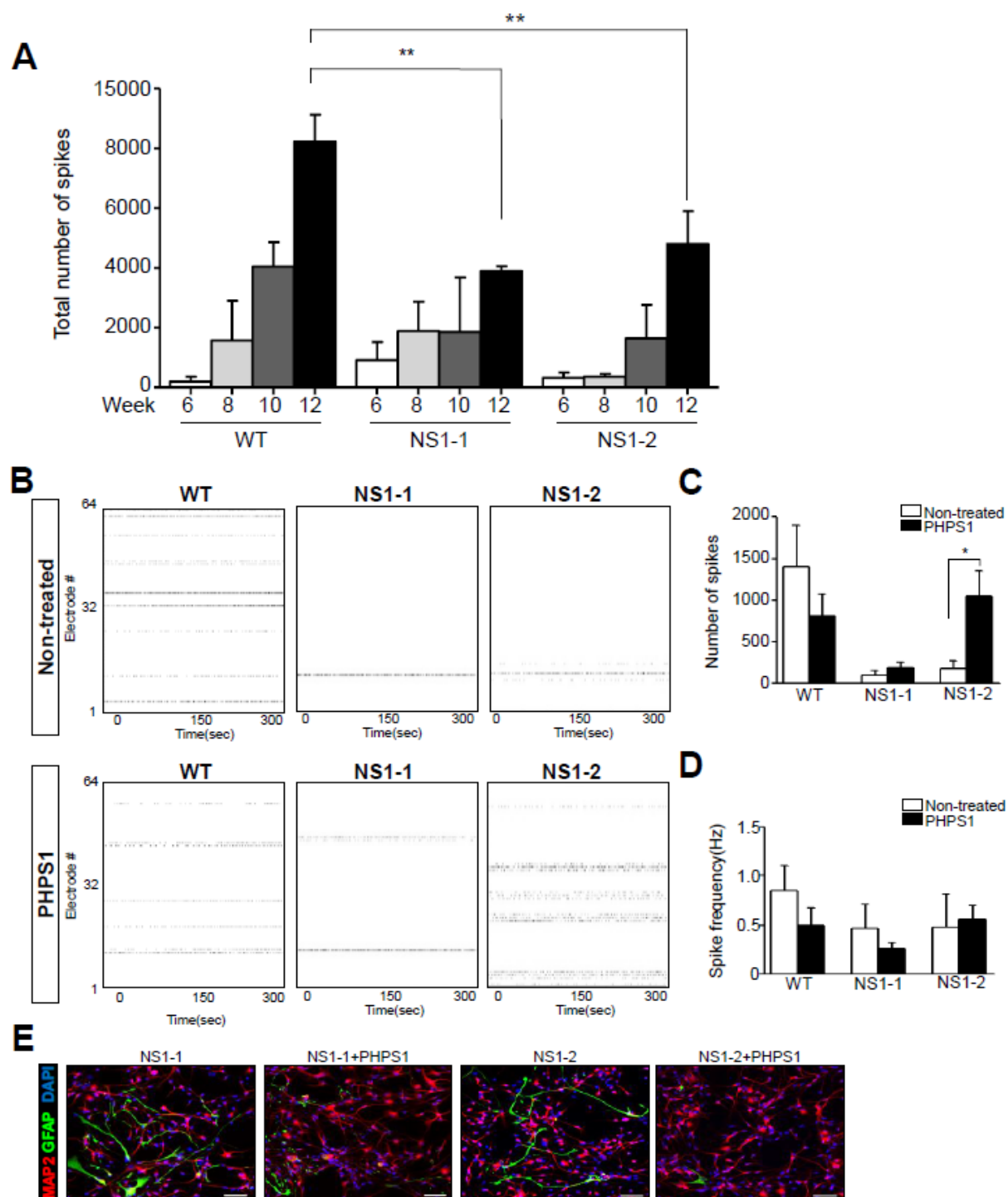

Supplement: Supplementary file 8 — Additional file 8: Figure S7. Time-course extracellular neural activities of NS-neural cells. (A) Monitoring of number of spontaneous extracellular spikes during neural differentiation. In WT- and NS-neural cells, recording of extracellular neural activities were obtained for 5 min at a two-week interval from 6 to 12 weeks during neural differentiation from NPCs. The number of extracellular spikes was significantly reduced in NS-neural cells at 12 week. Data were represented as mean ± SEM (6 week, n=5; 8 week, n=3; 10 week, n=2; 12 week, n=2). (B) Raster plot of extracellular spikes of NS-neural cells cultured for 12 weeks. Active channels with extracellular spikes in NS-neural cells were fewer than WT-neural cells (non treated, upper panel). A number of active channels increased via SHP2 inhibition in NS neural cells (lower panel). Extracellular spikes are shown as dots among the 64 electrodes. The bin size is 1 ms. The electrodes with extracellular spikes are defined as active channels. (C) Comparison of number of extracellular spikes between non- and PHPS1-treated groups. In the non-treated group (open bar), a small number of extracellular spikes were detected in NS-neural cells compared to WT-neural cells. SHP2 inhibition partially increased number of extracellular spikes in NS-neural cells (filled bar). (D) Spike frequency of NS-neural cells. No difference was detected in the spike frequency of neural cells between non- and SHP2 inhibited groups. (E) Decrease of glial cells in NS-neural cells after SHP2 inhibition. Glial cells were reduced in NS-neural cells after treatment of PHPS1. MAP2-positive (red) and GFAP-positive (green) cells represented neuronal and glial cells, respectively. Scale bar, 50 μm. These results were repeated twice independently with a different set of neural cells. P values were determined by using an unpaired Student’s t-test. *, p < 0.05; **, p < 0.01. [file 13287_2020_1709_MOESM8_ESM.pdf]

## Additional file 9: Figure S8

**A**

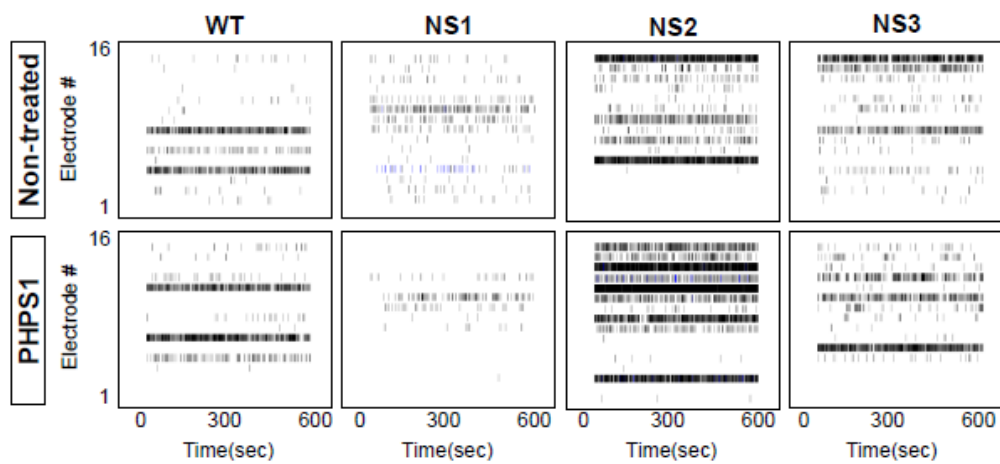

**B**

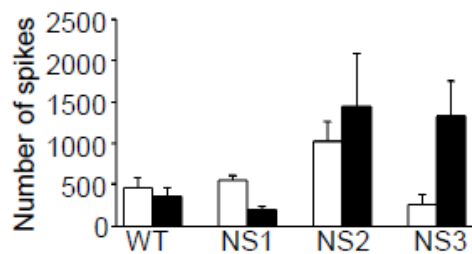

**C**

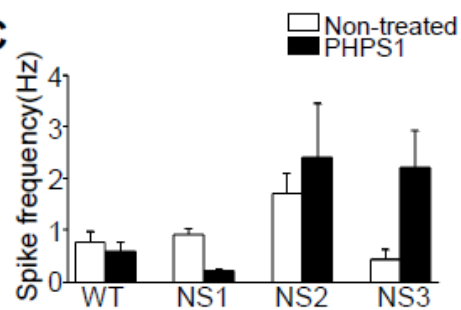

Supplement: Supplementary file 9 — Additional file 9: Figure S8. Spontaneous neural activities of cerebral organoids at 55 day of culture (A) Raster plots for extracellular spikes measured in cerebral organoids at 55 day of culture. (B-C) Number of spikes and spike frequency in cerebral organoids. These results were repeated from independently generated cerebral organoids (n = 3). [file 13287_2020_1709_MOESM9_ESM.pdf]

## Additional file 10: Fig. S9

**A**

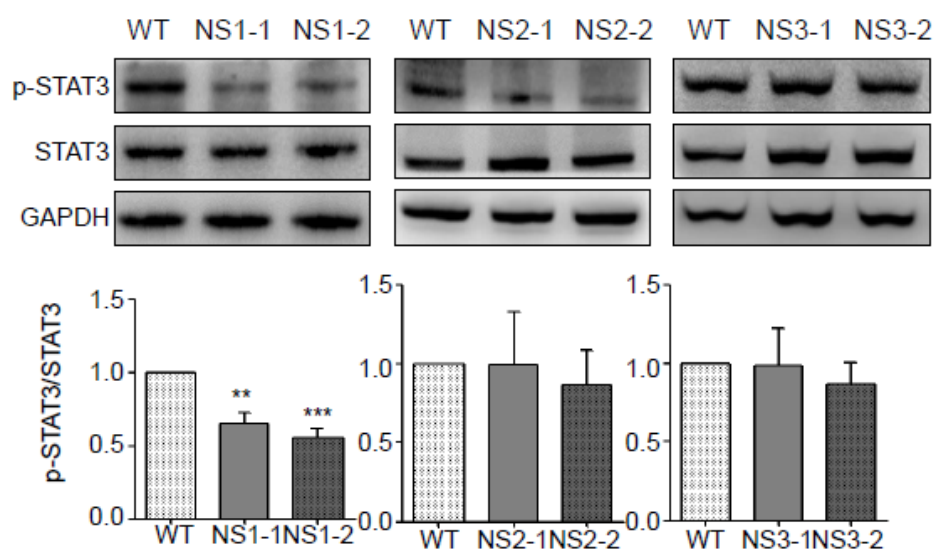

**B**

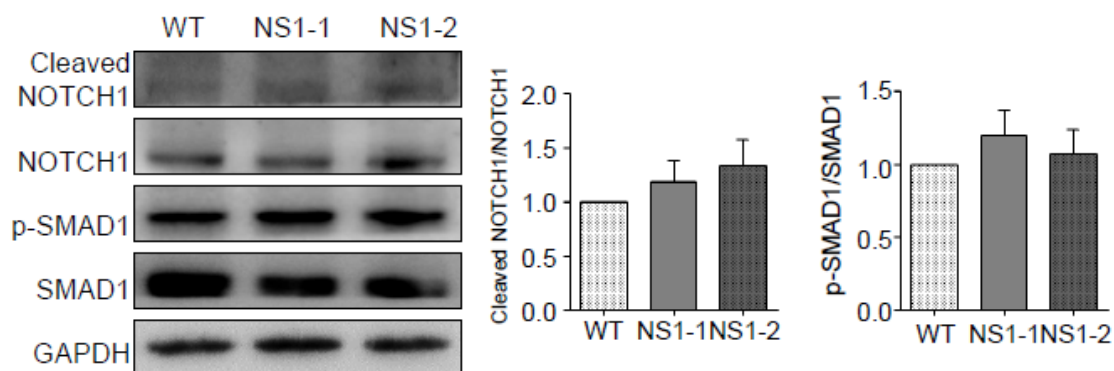

Supplement: Supplementary file 10 — Additional file 10: Figure S9. Activities of STAT3, NOTCH and BMP signalings in NS NPCs (A) Activity of p-STAT3 in the NS-NPCs. Level of p-STAT3 in NS-NPC was lower than WT ones. (B) Levels of cleaved NOTCH and p-SMAD1 in NS-NPCs. There was no difference in the level of cleaved NOTCH and p-SMAD1 between WT- and NS-NPCs. The relative band intensities are presented as the mean ± SEM (n=4). P values were determined by using an unpaired Student’s t-test. **, p < 0.01; ***, p < 0.001. [file 13287_2020_1709_MOESM10_ESM.pdf]
